# Supplementary figures and images for: Host phenotype classification from human microbiome data is mainly driven by the presence of microbial taxa
Source: PLoS Comput Biol. 2022 Apr 21;18(4):e1010066. doi: 10.1371/journal.pcbi.1010066 (PMC9064115; doi:10.1371/journal.pcbi.1010066)

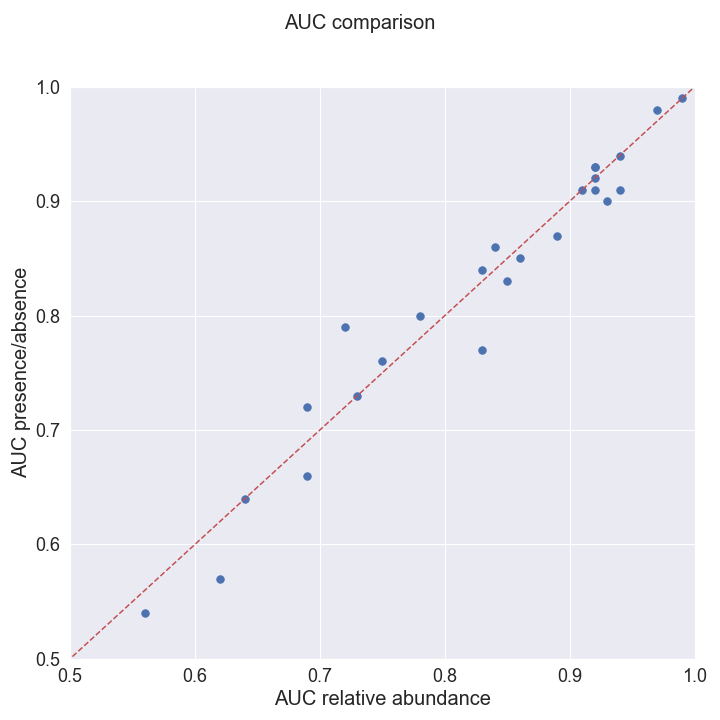

Supplement: S1 Fig — Comparison in terms of AUC between presence/absence and relative abundance profiles for the 25 case-control shotgun datasets. (PNG) [file pcbi.1010066.s014.png]

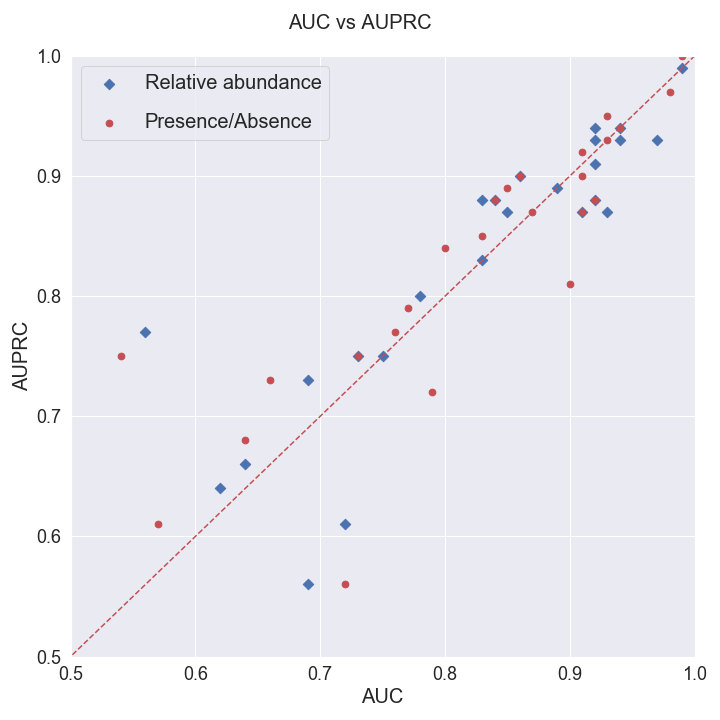

Supplement: S2 Fig — Comparison in terms of classification accuracies between AUC (area under the curve) and AUPRC (area under the precision-recall curve) for the 25 case-control shotgun datasets and by considering relative abundance (in blue; Spearman correlation = 0.889) and presence/absence (in red; Spearman correlation = 0.918) profiles. (PNG) [file pcbi.1010066.s015.png]

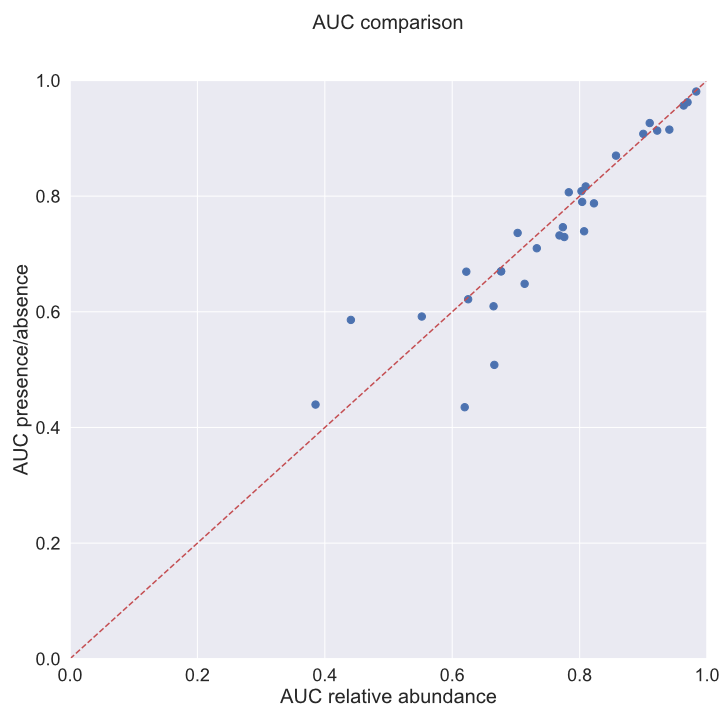

Supplement: S4 Fig — Comparison in terms of AUC between presence/absence and relative abundance profiles for the 30 case-control 16 rRNA datasets. (PNG) [file pcbi.1010066.s017.png]

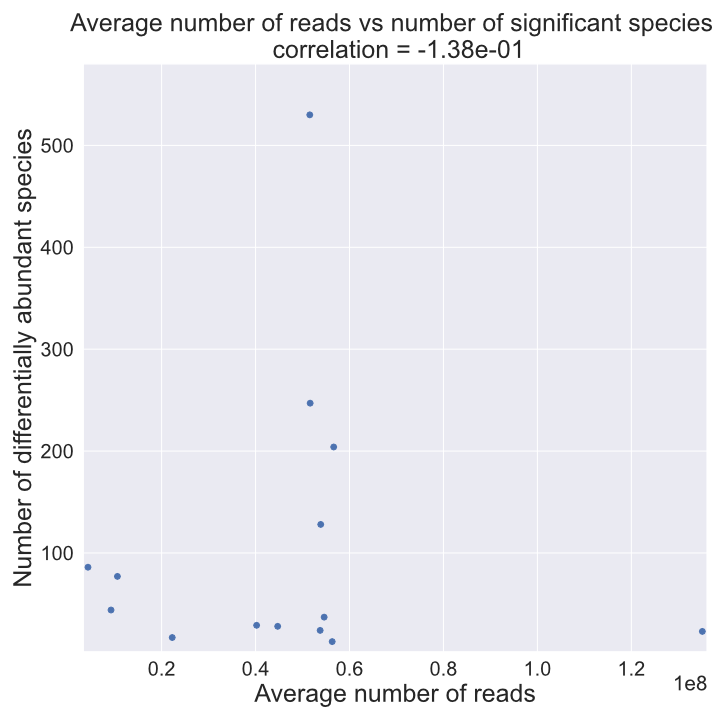

Supplement: S5 Fig — Each dot represents one of the 26 case-control shotgun studies. The number of statistically significant species is computed on relative abundance profiles. (PNG) [file pcbi.1010066.s018.png]

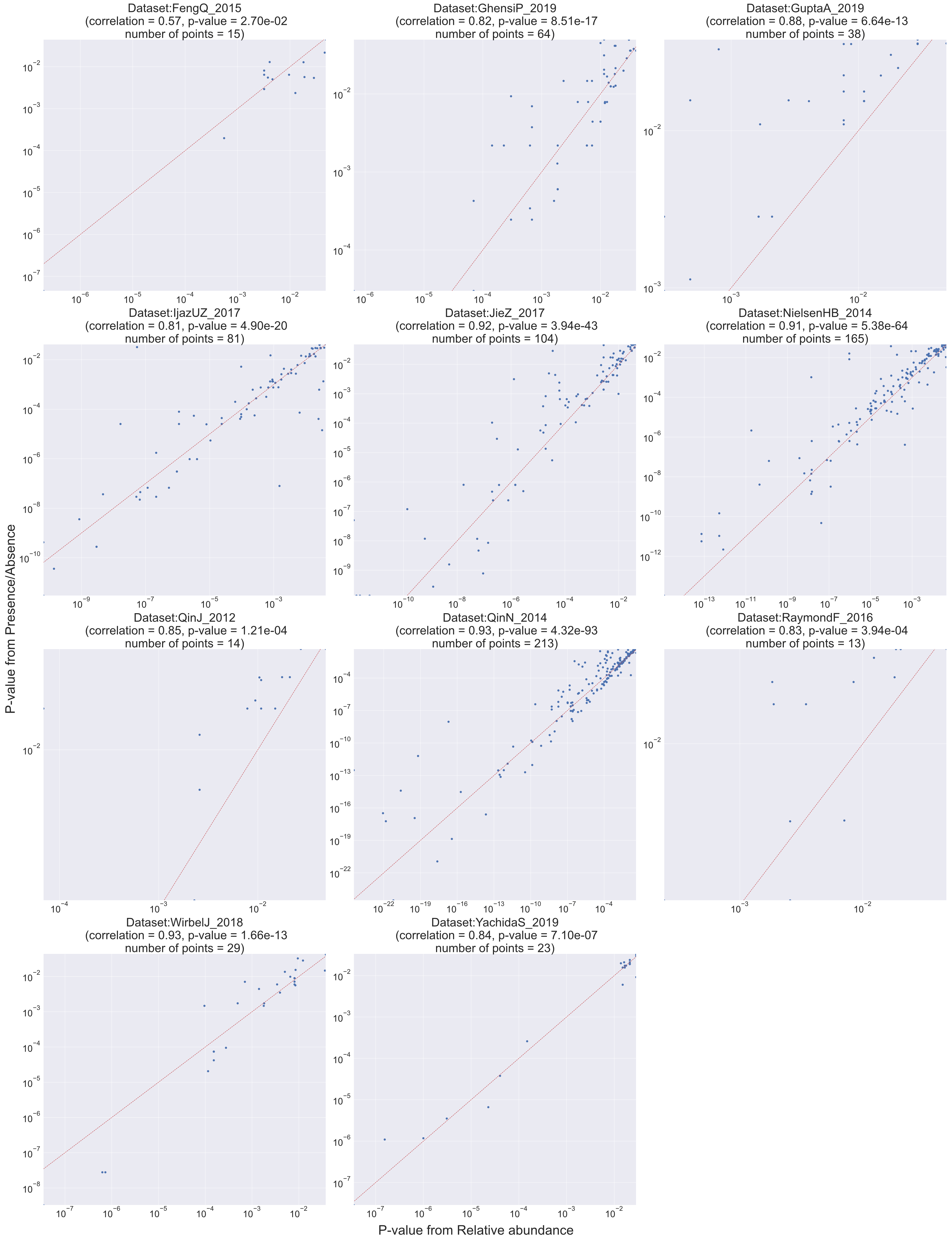

Supplement: S6 Fig — Each dot represents a different taxa (i.e., species) and we report only species significant in at least one of the two data types. Only datasets with at least ten data points are shown. (PNG) [file pcbi.1010066.s019.png]

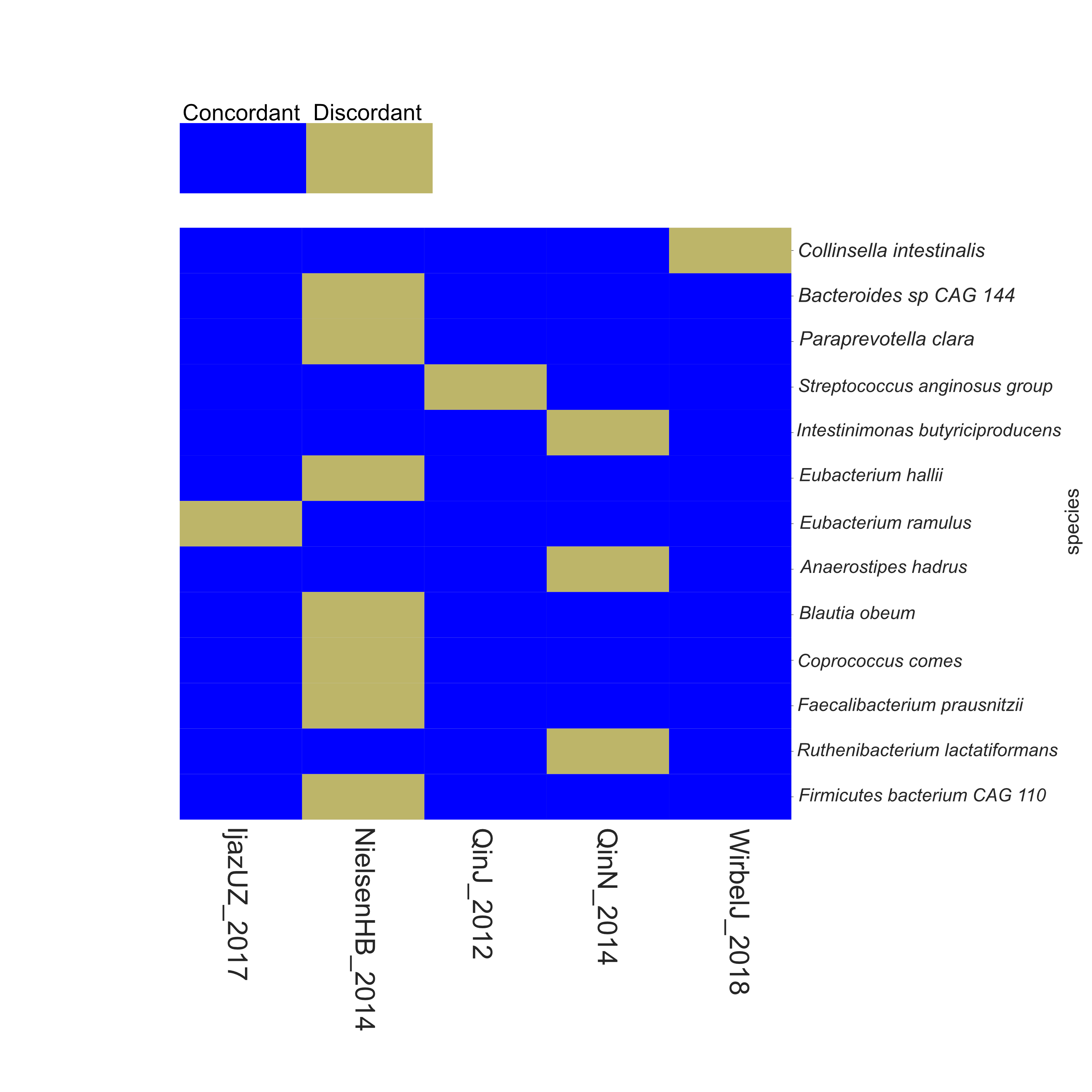

Supplement: S8 Fig — We identified discrepancies between case-enriched and control-enriched taxa derived from relative abundance and presence/absence data in only 1.74% of the statistically significant features, which were coming from just 5 datasets. No taxa disagreed across datasets. (PNG) [file pcbi.1010066.s021.png]

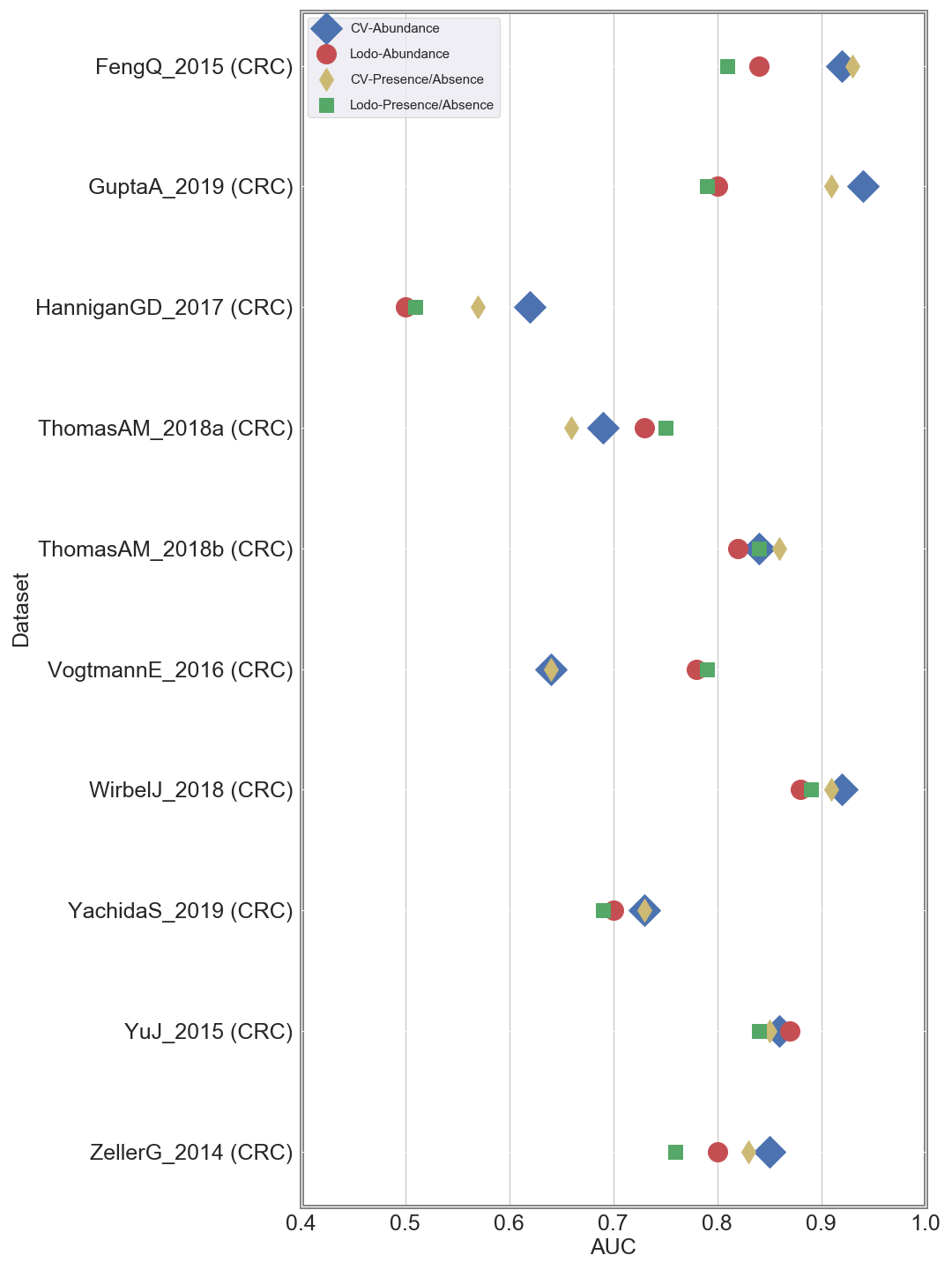

Supplement: S9 Fig — AUC scores using RF as back-end classifiers on species-level relative abundance and corresponding presence/absence profiles in CV and LODO settings. (PNG) [file pcbi.1010066.s022.png]

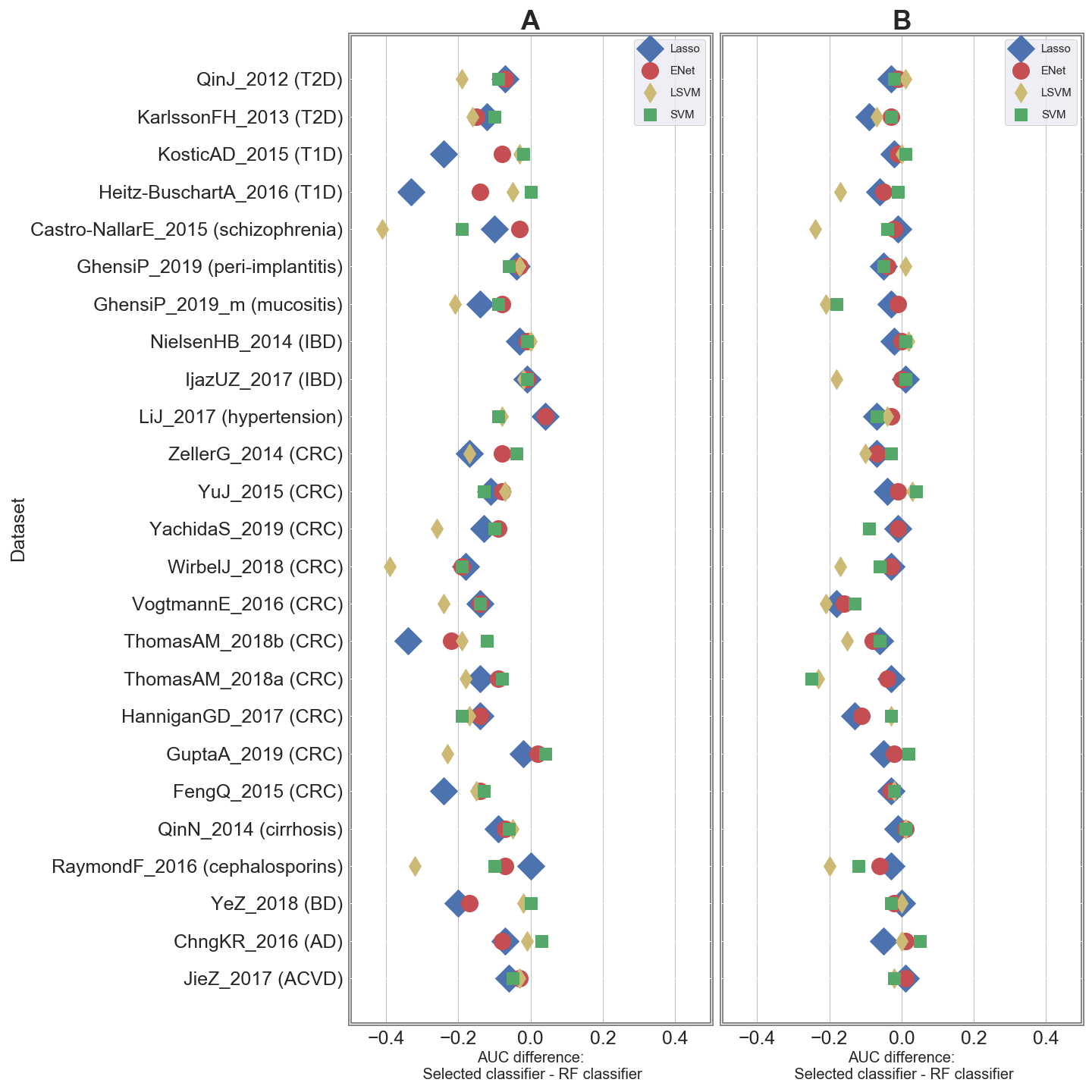

Supplement: S10 Fig — Results on the 25 case-control shotgun studies by considering different classification algorithms. Difference in AUC between RFs and other classification methods on (A) the relative abundance and (B) the presence/absence profiles. A positive value indicates that the comparison method outperforms RFs. (PNG) [file pcbi.1010066.s023.png]
