# Supplementary material for: Host phenotype classification from human microbiome data is mainly driven by the presence of microbial taxa
Source: PLoS Comput Biol. 2022 Apr 21;18(4):e1010066. doi: 10.1371/journal.pcbi.1010066 (PMC9064115; doi:10.1371/journal.pcbi.1010066)
Supplement: Attachment — Submitted filename: Presence absence meta analysis - Rebuttal letter.pdf [file pcbi.1010066.s024.pdf]

Editors

As you can see from the reviews, the feedback is generally positive and, in our judgement, none of the raised issues present a fundamental objection to the soundness and relevance of the work. We nonetheless ask that the authors consider all the points raised to improve the manuscript.

Response

We thank the Editors and the Reviewers for the overall positive feedback about our work. We feel that we addressed major points in this revised version.

Reviewer #1

General:

Giliberti and colleagues conduct a machine learning meta-analysis including different metagenomic datasets, using the information about presence and absence of bacterial species as input features for modelling rather than their relative abundance. The machine learning analyses are performed in a technically thorough manner and uncover a surprising finding, i.e. that the presence/absence information alone seems to be as predictive for various disease states as relative abundances. However, the biological relevance of the presented results are not fully clear and should be discussed in more detail.

Response

We thank the Reviewer for the overall appreciation of our work.

Major:

1. The authors discuss the implications for practical applications in diagnostic tests. However, it remains unclear to this referee whether detection of bacteria present rather than their quantification (by a cheap target approach such as qPCR) would make a diagnostic routine easier to implement in practice. The authors might want to discuss for which diagnostic assays this could matter.

Response

We agree that this discussion was not clear. We modified the last paragraph of the "Conclusions" section as follows (line 393):

The growing literature aiming at identifying microbial biomarkers for different diseases opened the possibility to build non-invasive diagnostic tools from microbiome data. To this purpose, much superior accuracy can be achieved by considering multi-feature rather than single biomarkers diagnostic models, and in which machine learning-based classification approaches have a fundamental role in building such models. Moreover, maximal accuracy can usually be achieved by using a limited number of features (in the order of ten or twenty). Such findings recently presented in the literature in addition to outcomes of our study, which suggest that the detection of microbial taxa is sufficient to maximize classification accuracies, are important steps toward the development of fast and inexpensive tests applied on stool samples for diagnostic purposes.

2. We feel that some of the chosen visualizations are not optimal to convey differences between various approaches in terms of AUC differences. A better alternative might be scatter plots with the original AUC on the x-axis and the AUC from the model trained on presence/absence data (or with different threshold, different taxonomic levels, or different machine learning algorithms) on the y-axis.

Response

As suggested by the Reviewer we added scatterplots that convey differences among various approaches in a better way. We added these plots as supplementary material, and more specifically as **S1 Fig** and **S3 Fig** for the shotgun data and as **S4 Fig** for the 16S data:

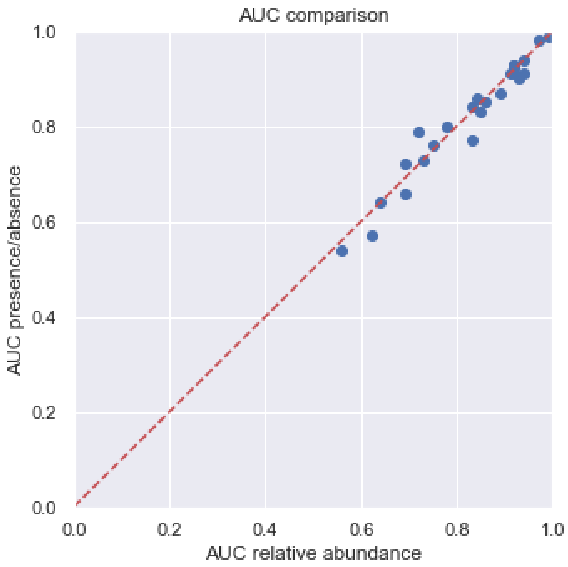

S1 Fig. Classification accuracies are robust to degradation from species-level relative abundance to presence/absence profiles in shotgun datasets. Comparison in terms of AUC between presence/absence and relative abundance profiles for the 25 case-control shotgun datasets.

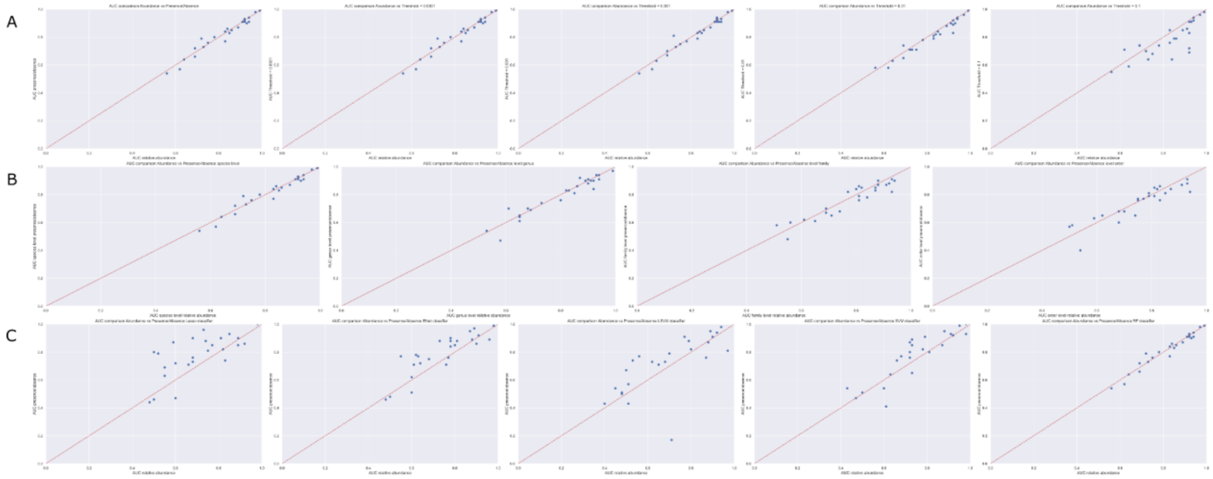

S3 Fig. Classification accuracies are robust to degradation from species-level relative abundance to presence/absence profiles in shotgun datasets. Comparison in terms of AUC between presence/absence and relative abundance profiles for the 25 case-control shotgun datasets by (**A**) thresholding at different relative abundance values (ranging from 0% to 0.1%), (**B**) changing taxonomic resolution (from species to order level), and (**C**) changing classification algorithm.

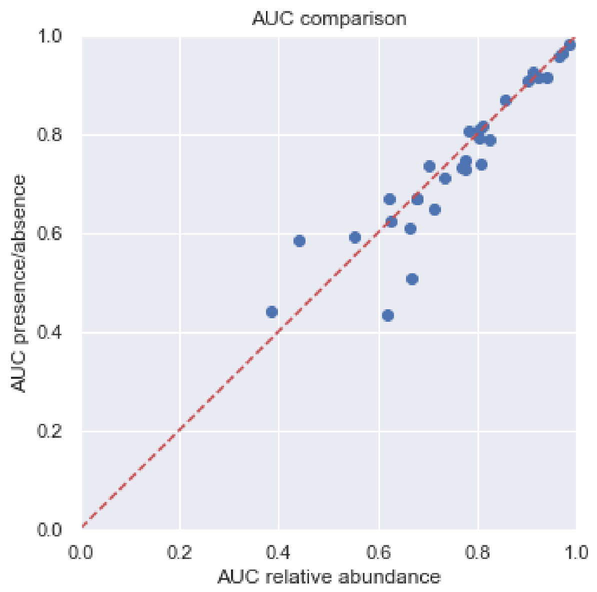

S4 Fig. Classification accuracies are robust to degradation from species-level relative abundance to presence/absence profiles in 16S rRNA datasets. Comparison in terms of AUC between presence/absence and relative abundance profiles for the 30 case-control 16 rRNA datasets.

And we modified the main text accordingly in multiple parts (line 244):

Surprisingly, we observed negligible differences between the two experimental settings (**Fig 1B, Fig 1C, S1 Fig**, and **S2 Table**).

and (line 311):

We did not observe changes in the classification accuracy when the threshold was set to 0.0001% and 0.001% (**Fig 3A, S3 Fig part A**, and **S2 Table**).

and (line 338):

While no differences were obtained at species-level (as already discussed in **Fig 1**), we observed that coarser resolutions brought increasing AUC differences (**Fig 4**, **S3 Fig part B**, and **S11 Table**).

and (line 362):

On average, thresholding of relative abundance values did not negatively impact classification accuracies, instead it generally improved results in a quite unexpected way (**Fig 5** and **S3 Fig part C**).

and (line 265):

By degrading relative abundance to presence/absence profile, we obtained few differences in the classification results between the two profile types (**Fig 2B, Fig 2C**, **S4 Fig** and **S5 Table**).

3. The included datasets (and samples within a dataset) vary considerably in terms of their mean sequencing depth, which likely affects the detection of taxa with lower relative abundance. For this reason data is often downsampled (rarefied) before presence/absence calls and derived richness estimates are calculated. This issue is partially addressed by the authors' use of different threshold values above which the authors call a taxa "present", but this aspect might need further clarification. A more explicit description of how their thresholding strategy relates to downsampling datasets should be added and in the best case show both approaches could be compared empirically (on a few datasets).

Response

First, we assessed to what extent there was a correlation between the number of statistically significant species and the average number of reads on a per dataset basis. We didn't find any correlation as summarized in the **S5 Fig**:

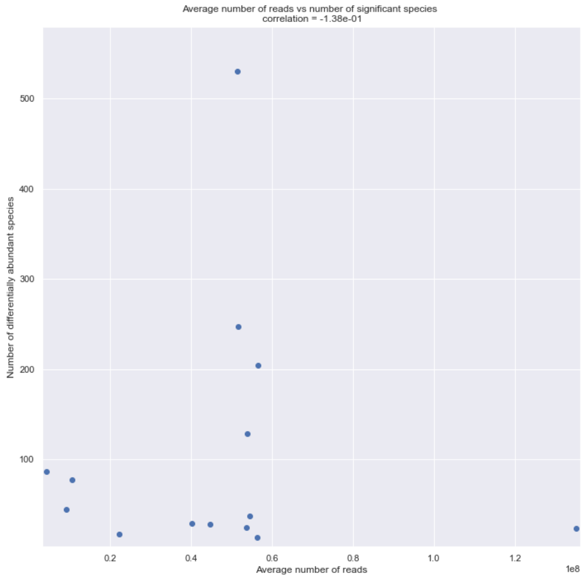

S5 Fig. Number of differentially abundant species has weak correlation with the average number of reads. Each dot represents one of the 26 case-control shotgun studies. The number of statistically significant species is computed on relative abundance profiles.

And discussed in the text as follows (line 275):

By comparing the sets of statistically significant species in the different case-control studies (q < 0.05; using Mann-Whitney U test for relative abundance and Fisher exact test for presence/absence profiles, both corrected through false detection rate (FDR), **S6 Table**) we found similar numbers (**Fig 1D** and **S7 Table**), with values more driven by disease and dataset types than average number of reads (**S5 Fig**).

We further conducted a rarefaction analysis that is described in the "Materials and Methods" section (new subsection "Rarefaction analysis") as follows (line 205):

We further performed rarefaction analysis by: i) considering the three datasets having the highest number of significant species from relative abundance profiles (i.e., JieZ_2017, NielsenHB_2014, and QinN_2014); ii) rarefying raw reads (using https://github.com/lh3/seqtk) and considering 1M reads for each metagenome; iii) applying the same pipeline to generate taxonomic profiles through MetaPhlAn3; iv) applying the same pipeline to build classification models and identifying statistically significant species.

Results are summarized in the new **S9 Table**:

S9 Table. Results obtained on three selected shotgun datasets after rarefying metagenomes at 1M reads. Comparison in terms of AUC, F1, precision, recall, in addition to number of statistically significant taxa (q<=0.05), between the results obtained classifying on the abundances matrix and the classification made on the presence/absence boolean matrix at different taxonomic levels (only at species level).

And discussed in the text as follows (line 324):

Results on rarefied reads (**Methods**) showed, as expected, a slight decrease in terms of classification accuracies and number of detected biomarkers with respect to the original data set, although patterns in function of the thresholding value when going from relative abundance to presence/absence data were confirmed (**S9 Table**).

4. The analysis of statistically significant taxa in both approaches (lines 330 and following, also SFig. 4) feels disconnected from the rest of the manuscript and generally underdeveloped.

Response

We moved this part earlier in the manuscript and put it in the new section "Statistically significant taxa are consistent between relative abundance and presence/absence profiles". We also performed additional analyses and added more text as follows (line 272):

Statistically significant taxa are consistent between relative abundance and presence/absence profiles

We extended the analysis from classification to identification of differentially abundant/present taxa (i.e., possible biomarkers) through statistical testing (**Methods**). By comparing the sets of statistically significant species in the different case-control studies ($q < 0.05$; using Mann-Whitney U test for relative abundance and Fisher exact test for presence/absence profiles, both corrected through false detection rate (FDR), **S6 Table**) we found similar numbers (**Fig 1D** and **S7 Table**), with values more driven by disease and dataset types than average number of reads (**S5 Fig**). On average, we found 39 and 32 significant species from relative abundance and presence/absence profiles, respectively.

On a per dataset basis, p-values associated with statistically significant species correlated well between relative abundance and presence/absence profiles (**S6 Fig**). This was reflected also by the high percentage of taxa (78%) that were detected as significant in both cases, which was further confirmed by performing hierarchical clustering on the set of statistically significant taxa coming from relative abundance and presence/absence profiles (**S7 Fig**). Conversely, we identified discrepancies between case-enriched and control-enriched taxa in only 1.74% of the statistically significant features, which were coming from just 5 of the 24 analysed datasets (**S8 Fig**). Moreover, we didn't identify any taxa for which the two tests disagreed across datasets (**S8 Fig**).

Focusing on the gut microbiome datasets, we also identified the species that were mostly associated with disease or health (**S7 Fig**). The species most enriched in cases was *Clostridium bolteae* (significant in 78% of the diseases), followed by *Streptococcus anginosus group* (55%), *Ruthenibacterium lactatiformans* (55%), *Hungatella hathewayi* (55%), and *Eisenbergiella tayi* (55%) with all of them already reported in the literature as possible biomarkers for different disease conditions [6,13,39,41,56]. Similarly, species most enriched in controls were *Anaerostipes hadrus* (significant in 66% of the diseases), *Roseburia faecis* (55%), *Roseburia intestinalis* (55%), *Prevotella copri* (44%), and *Eubacterium hallii* (44%) [6,10,39,57].

Consistence between relative abundance and presence/absence outcomes was finally obtained on the 16S data, with 20 and 15 genera that were found to be significant on average from relative abundance and presence/absence profiles, respectively (**Fig 2D** and **S8 Table**).

Could the authors compare the p-values from the Wilcoxon and Fisher tests, maybe again via scatter plots (on -log10 scale of the p-values)? How well do the p-values correlate?

<u>Response</u>

We performed this analysis and obtained high correlations between p-values from the Wilcoxon and Fisher tests. We added this as new **S6 Fig**:

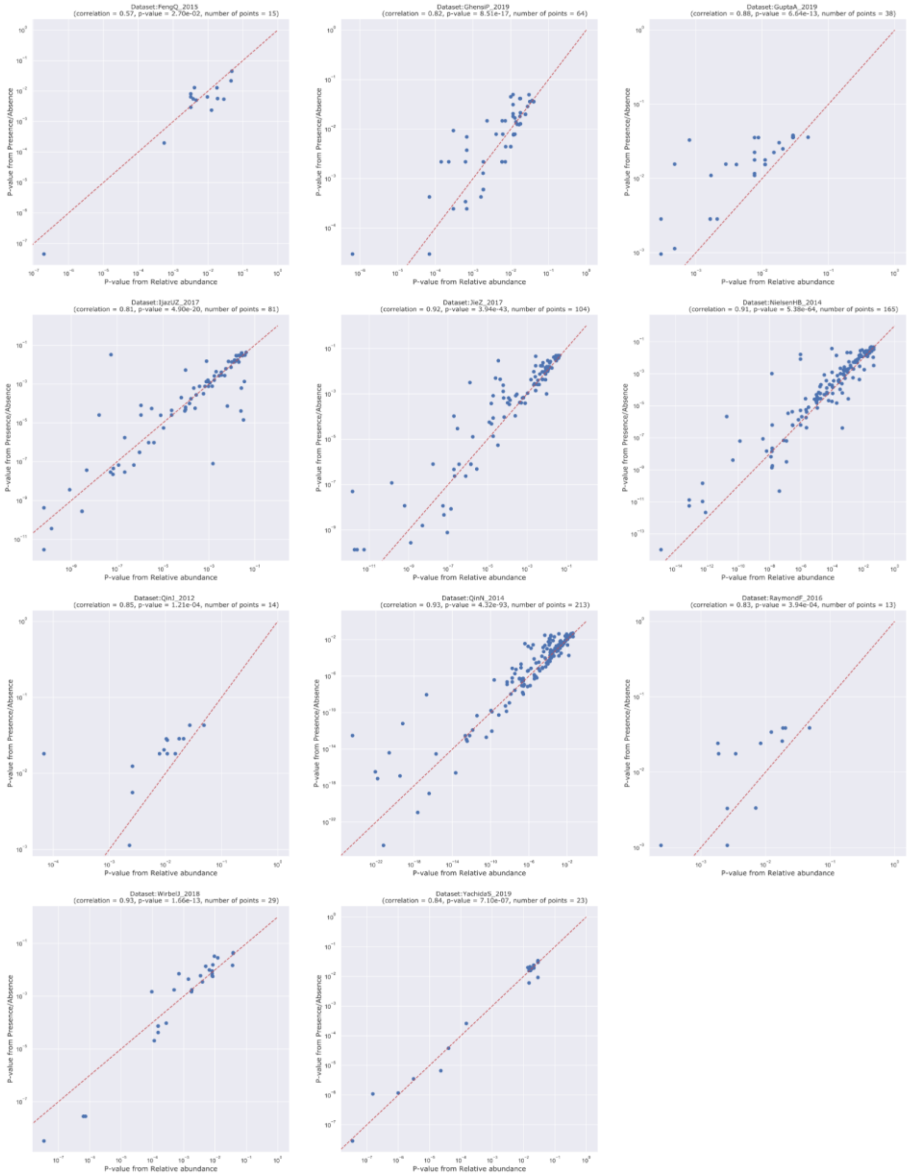

S6 Fig. P-values associated with statistically significant species correlate well between relative abundance and presence/absence profiles. Each dot represents a different taxa (i.e., species) and we report only species significant in at least one of the two data types. Only datasets with at least ten data points are shown.

And discussed this in the text (line 285):

On a per dataset basis, p-values associated with statistically significant species correlated well between relative abundance and presence/absence profiles (**S6 Fig**).

Are there taxa for which the two tests disagree consistently across datasets?

Response

This aspect was addressed only marginally in the previous version of the manuscript, and expanded more now. We summarized results in the new **S8 Fig**:

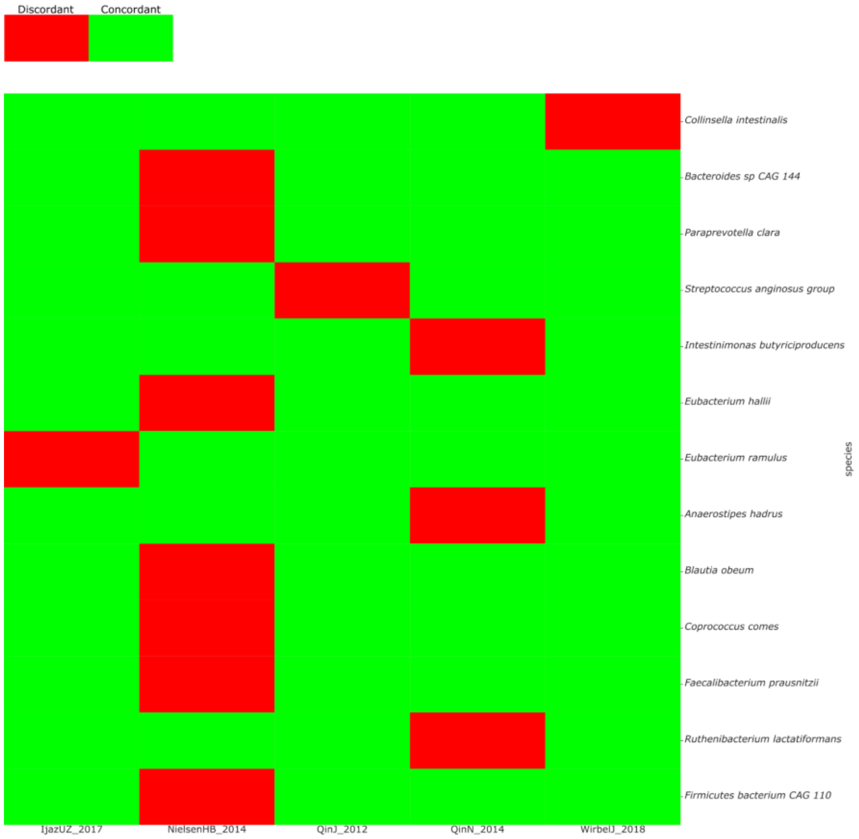

S8 Fig. Statistically significant taxa from relative abundance and presence/absence profiles did not disagree across datasets. We identified discrepancies between case-enriched and control-enriched taxa derived from relative abundance and presence/absence data in only 1.74% of the statistically significant features, which were coming from just 5 datasets. No taxa disagreed across datasets.

And discussed this in the text (line 289):

Conversely, we identified discrepancies between case-enriched and control-enriched taxa in only 1.74% of the statistically significant features, which were coming from just 5 of the 24 analysed datasets (**S8 Fig**). Moreover, we didn't identify any taxa for which the two tests disagreed across datasets (**S8 Fig**).

How does it look like when abundance fold change and prevalence difference across groups is compared? Is there a clear correlation? In general, this analysis could be greatly expanded to bolster the biological interpretation of the results.

Response

We thank the Reviewer for the nice suggestion. We performed some analyses in this direction, however we didn't find any interesting pattern to show and discuss in the manuscript.

In this context, it is also important to note that there are many CRC datasets in the set of included studies, potentially biasing the disease-associations reported in lines 339 and following. Instead, the

authors could try to get a single significance value for each disease (including all available studies of this disease) and compare again.

<u>Response</u>

We perfectly agree on this. We redid the analysis on a per disease instead of a per dataset basis. We updated the **S7 Fig**:

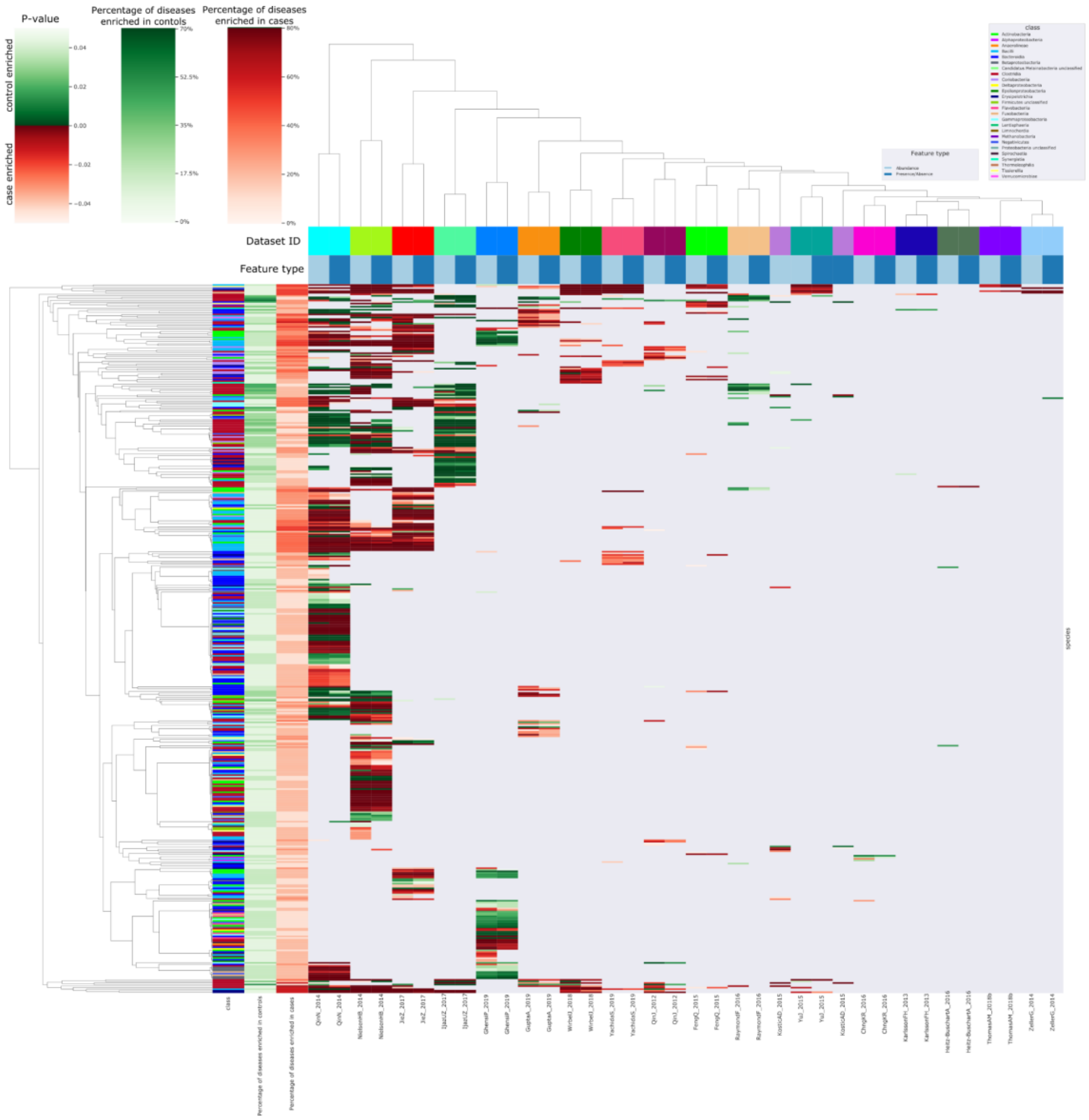

S7 Fig. Statistically significant taxa are consistent between relative abundance and presence/absence data on a per dataset basis. Heatmap generated on the p-values (after FDR correction; $p > 0.05$ in grey) obtained by applying statistical tests on the case-control metagenomic datasets. Only the 18 datasets with at least one discriminative taxa are reported. Left-most colorbar identifies the taxonomic class of each taxa. The two right-most colorbars indicate the percentage of diseases for which the species resulted to be enriched in controls (in green) and in cases (in red). This percentage is computed on a per disease basis, when multiple datasets are available for the same disease, the taxa is considered significant when detected as significant in at least one dataset.

And the text accordingly (line 286):

This was reflected also by the high percentage of taxa (78%) that were detected as significant in both cases, which was further confirmed by performing hierarchical clustering on the set of statistically significant taxa coming from relative abundance and presence/absence profiles (**S7 Fig**). [...]
Focusing on the gut microbiome datasets, we also identified the species that were mostly associated with disease or health (**S7 Fig**). The species most enriched in cases was *Clostridium bolteae* (significant in 78% of the diseases), followed by *Streptococcus anginosus group* (55%), *Ruthenibacterium lactatiformans* (55%), *Hungatella hathewayi* (55%), and *Eisenbergiella tayi* (55%) with all of them already reported in the literature as possible biomarkers for different disease conditions [6,13,39,41,56]. Similarly, species most enriched in controls were *Anaerostipes hadrus* (significant in 66% of the diseases), *Roseburia faecis* (55%), *Roseburia intestinalis* (55%), *Prevotella copri* (44%), and *Eubacterium hallii* (44%) [6,10,39,57].

5. Does the disease which is to be predicted have any influence on loss of/retaining accuracy after converting features to presence/absence information? One could imagine that CRC, relying more on rare biomarkers, could be less affected by downgrading features to presence/absence information than for example IBD, which is characterized by stronger community shifts in highly abundant and prevalent taxa. This analysis could contribute to developing a clearer understanding of the biological relevance of the presented analysis.

Response
This is an interesting question. We agree with the hypothesis formulated by the Reviewer, although there was weak evidence based on our data and results. We added few sentences in the text as follows (line 280):
We may hypothesize that diseases that rely on rare biomarkers are less affected by degradation to presence/absence profiles than the ones that are characterized by stronger community shifts in abundant and prevalent taxa. Although this is not sufficiently supported by our data, further investigation in this direction is warranted.

Minor:
1. The LODO analysis for CRC studies could be moved from the supplement to the main manuscript, especially if the display items are streamlined via scatter plots.

Response
We agree on this and moved the analysis from the supplementary material to the main manuscript (as **Fig 6**):

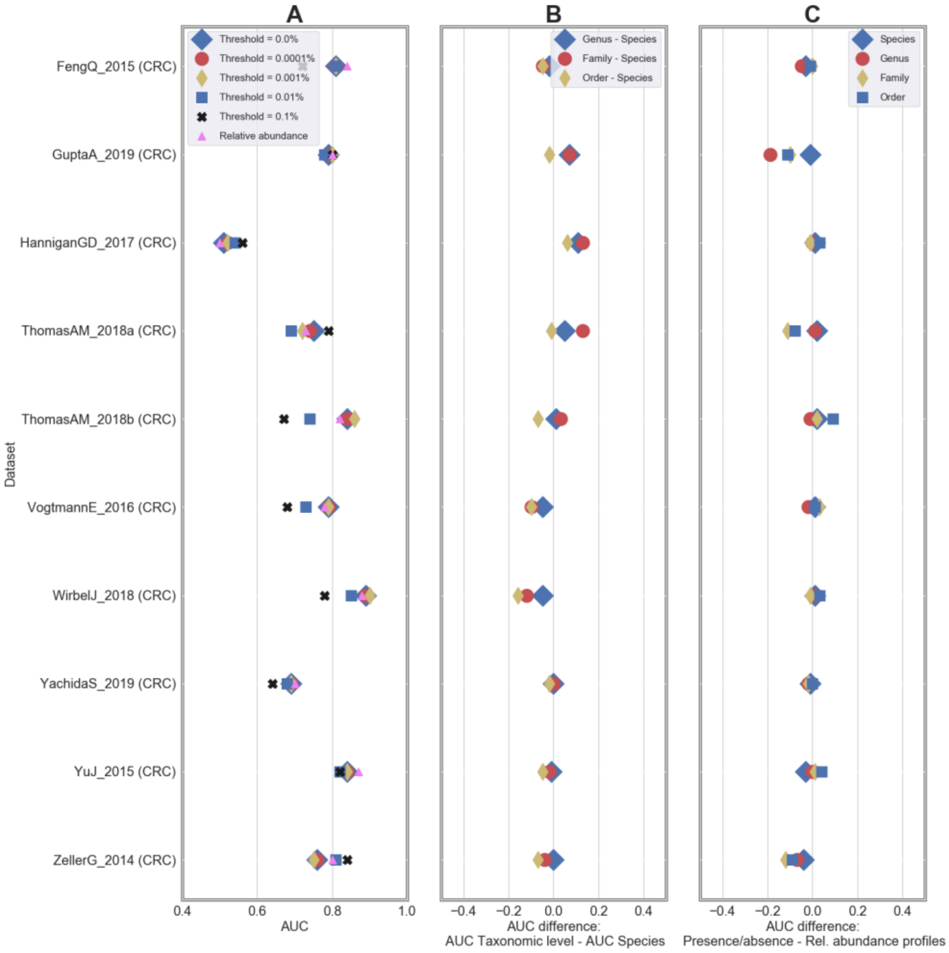

Fig 6. **Degradation of relative abundance profiles does not impact LODO classification**. Results in terms of leave-one-dataset-out (LODO) validation on 10 CRC shotgun datasets. (**A**) AUC scores using RF as back-end classifiers on species-level relative abundance (in pink) and presence/absence profiles generated at different threshold values. (**B**) Difference in AUC between species and other taxonomic-level resolutions. A negative value indicates that species-level outperforms the comparison level. (**C**) Difference in AUC between presence/absence and relative abundance classification results at varying taxonomic levels.

2. Are the different models trained on the same cross-validation splits? If not, adopting this approach could remove the random noise introduced by repeated CV splitting.

<u>Response</u>

Yes, different models were trained on the same cross-validation splits. This was actually an important detail that was missing and that we added in the “Validation and evaluation strategies” section (line 136):

In cross-validation, samples were randomly divided into *k* (with k = 10 in our case) folds by considering a stratified cross-validation approach to preserve the percentage of samples of each class. Results were repeated and averaged on 20 independent runs. Different models were trained on the same cross-validation splits.

Reviewer #2

Giliberti et al. present a very well written paper comparing the predictive performance of quantitative vs qualitative taxonomic profiles. The breadth of the work is comprehensive in the range of phenotypes (IBD, CRC, T2D, etc) being analyzed and the data types (16S rRNA and Metagenomics). The core question being answered is an interesting one and this paper will likely be of interest to the broad microbiome community. However, there are three main issues that would first need to be addressed. The first is a logistical issue concerned with the availability of the source code used to generate the data in this paper. The second issue is concerned with the use of the AUC of the ROC to evaluate classification tasks on unbalanced datasets. The last issue is concerned with the choice of test used to compute differential abundance.

Response

We thank the Reviewer for the overall appreciation of our work. Very briefly, we addressed the three raised issues as follows, with more details in the next responses: i) we made a public repository hosted in GitHub with a tutorial that goes through the entire analysis pipeline and all the data and code necessary to reproduce the results and figures/tables of the paper; ii) we complemented AUC evaluation with AUPRC values, which were in strong agreement; iii) we kept the statistical tests that were considered in the previous version of the manuscript, however some additional evaluations will be discussed in this letter and some discussions were added to the main text.

The code used to generate the data and figures included in this paper needs to be made available and well documented. Please note the versions of the software dependencies used, how to run these scripts, and when and where the datasets were downloaded from. My recommendation is to host this repository on GitHub or another source code sharing platform.

Response

We created a public repository hosted in GitHub (https://github.com/RGilib/giliberti-meta-analysis-2022) and more specifically a detailed Wiki section (https://github.com/RGilib/giliberti-meta-analysis-2022/wiki).

This Wiki is structured in two main sections. First, a tutorial on a single dataset aiming at going through the entire pipeline. Then, the data and code necessary to replicate all results and figures/tables of the paper. We also list in the Wiki the list of software/libraries with their versions.

We added this sentence in the Data availability statement:

The data and source code used to produce the results and analyses presented in this manuscript are available on a GitHub repository at https://github.com/RGilib/giliberti-meta-analysis-2022.

Using the AUC of the ROC for evaluating the performance of a classification task for unbalanced datasets is likely to yield misleading results. My suggestion would be to use this metric and the area under the precision-recall curve AUPRC. Alternatively using any of the other metrics that were already measured for this dataset would also be appropriate. These metrics need to be also evaluated with the statistical testing framework used for the AUC of the ROC.

Response

We complemented AUC evaluation with AUPRC values. Results were in strong agreement. We added these results in **Fig 1** (for shotgun) and **Fig 2** (for 16S) as a new panel C:

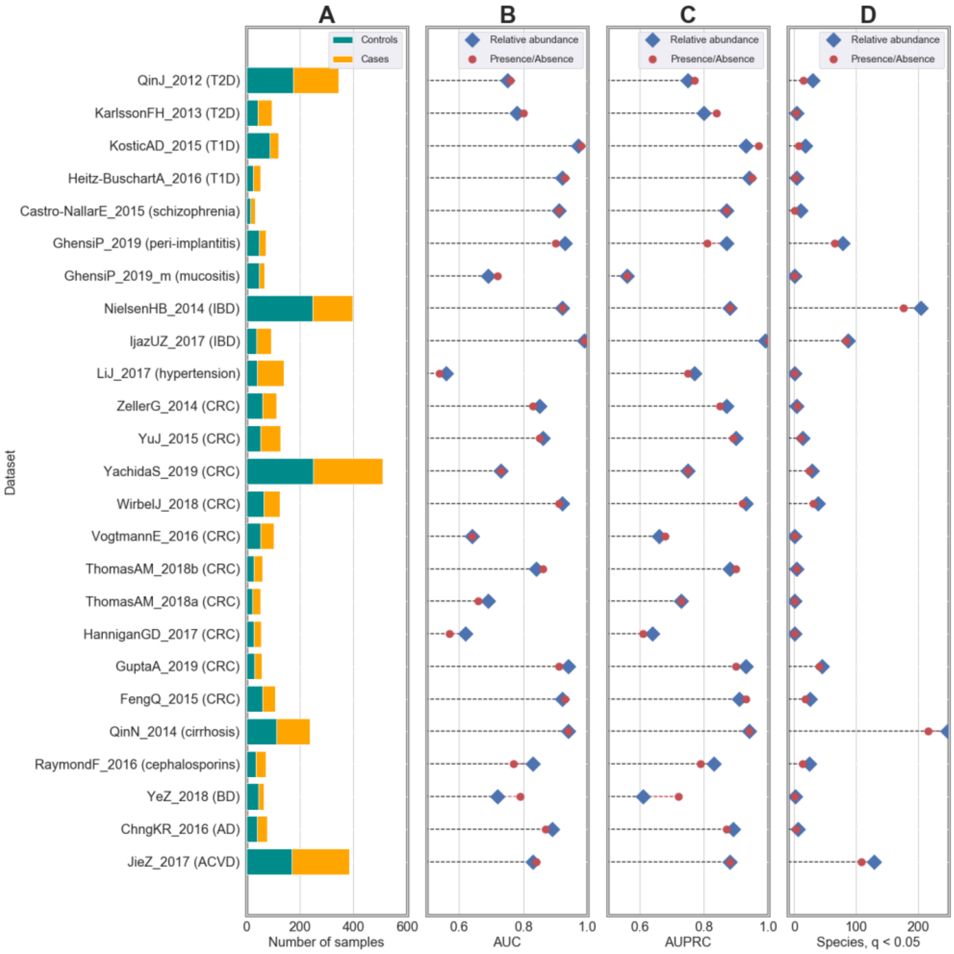

Fig 1. **Classification accuracies are robust to degradation from species-level relative abundance to presence/absence profiles in shotgun datasets.** Results obtained on 25 case-control studies for host phenotype classification from human microbiomes. (**A**) Number of case and control samples across the different studies. (**B**) AUC and (**C**) AUPRC scores using RF as back-end classifiers on species-level taxonomic profiles. Comparison between relative abundance (in blue) and presence/absence (in red) profiles highlighted negligible differences and no statistical differences in none of the studies (see **S2 Table** for p-values). Metrics of comparison in terms of AUC, AUPRC, precision, recall, and F1 are summarized in **S2 Table**. (**D**) Number of statistically significant taxa from relative abundance (in blue) and presence/absence (in red) profiles.

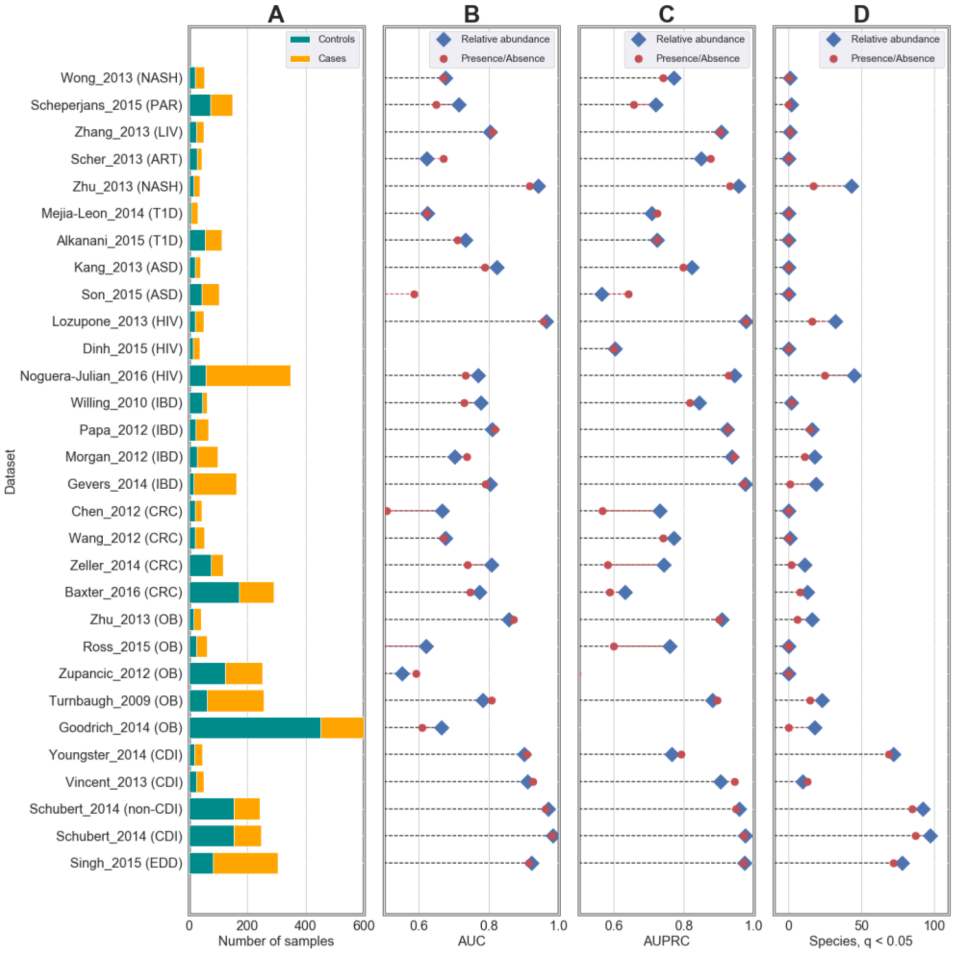

Fig 2. Classification accuracies are robust to degradation from genus-level relative abundance to presence/absence profiles in 16S rRNA datasets. Results obtained on 30 case-control studies for host phenotype classification from human microbiomes. (**A**) Number of case and control samples across the different studies. (**B**) AUC and (**C**) AUPRC scores using RF as back-end classifiers on species-level taxonomic profiles. Comparison between relative abundance (in blue) and presence/absence (in red) profiles highlighted negligible differences and no statistical differences in none of the studies (see **S5 Table** for p-values) as found also in shotgun datasets (see **Fig 1**). Metrics of comparison in terms of AUC, AUPRC, precision, recall, and F1 are summarized in **S5 Table**. (**C**) Number of statistically significant taxa from relative abundance (in blue) and presence/absence (in red) profiles.

Such AUPRC values were also added accordingly to **S2 Table** (for shotgun) and **S5 Table** (for 16S).

For shotgun data, we also produced a scatterplot showing the strong correlation between AUC and AUPRC values as new **S2 Fig**:

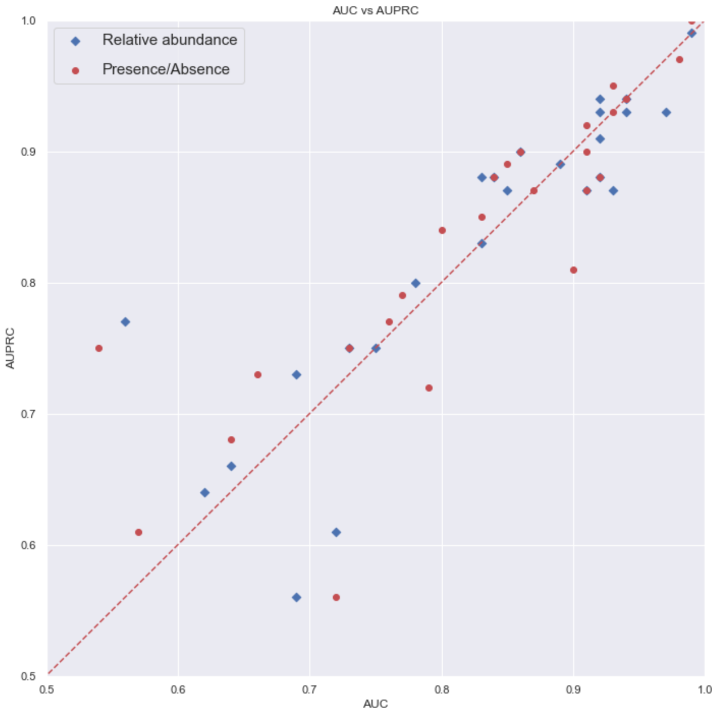

S2 Fig. AUC correlates well with AUPRC. Comparison in terms of classification accuracies between AUC (area under the curve) and AUPRC (area under the precision-recall curve) for the 25 case-control shotgun datasets and by considering relative abundance (in blue; Spearman correlation = 0.889) and presence/absence (in red; Spearman correlation = 0.918) profiles.

We commented these results in the text as follows (line 228):

More specifically, we considered a RF classifier applied on the species-level relative abundance profiles, and evaluated classification accuracies in terms of multiple metrics (i.e., area under the ROC curve (AUC), area under the precision-recall curve (AUPRC), precision, recall, and F1) using a cross-validation (CV) approach (see **Methods**).

and (line 244):

Surprisingly, we observed negligible differences between the two experimental settings (**Fig 1B, Fig 1C, S1 Fig**, and **S2 Table**). In both cases (i.e., using presence/absence or relative abundance profiles), we obtained an average AUC of 0.83 (AUPRC = 0.83) across the 25 case-control studies, with AUC and AUPRC values strongly correlated (**S2 Fig**; Spearman correlation = 0.918).

and (line 265):

By degrading relative abundance to presence/absence profile, we obtained few differences in the classification results between the two profile types (**Fig 2B, Fig 2C**, **S4 Fig** and **S5 Table**).

Lastly, performing differential abundance comparisons using Mann-Whitney's U test is inappropriate due to the compositional nature of the data. The authors should update these results to use a different test and or normalization strategy.

Response

We thank the Reviewer for raising this point. All sequencing data are compositional in nature. For RNA-seq data it has not proven to be a major consideration, and in limited benchmarking in microbiome data it is still not clear that it should be a primary consideration in all habitats. We also

conducted a sensitivity analysis using the recently proposed method ZINQ that takes into account the compositional issue for microbiome data analysis (Ling W, Zhao N, Plantinga AM, Launer LJ, Fodor AA, Meyer KA, et al. Powerful and robust non-parametric association testing for microbiome data via a zero-inflated quantile approach (ZINQ). Microbiome. 2021;9: 181.). Results in terms of statistically significant taxa (at species and genus level) are summarized in this table and show quite comparable numbers with what we report in the paper using the Mann-Whitney's U test:

**datasetID**	**Paper_species**	**ZINQ_species**	**Paper_genus**	**ZINQ_genus**
Castro-NallarE_2015	9	6	3	4
ChngKR_2016	5	3	3	1
FengQ_2015	24	19	24	13
GhensiP_2019	77	59	36	19
GhensiP_2019_m	0	3	0	1
GuptaA_2019	44	30	26	16
HanniganGD_2017	0	0	0	0
Heitz-BuschartA_2016	3	2	3	2
IjazUZ_2017	86	70	51	41
JieZ_2017	128	100	51	44
KarlssonFH_2013	3	0	0	0
KosticAD_2015	17	13	6	2
LiJ_2017	0	0	0	0
NielsenHB_2014	204	150	78	65
QinJ_2012	29	17	25	14
QinN_2014	247	186	83	72
RaymondF_2016	23	15	13	8
ThomasAM_2018a	0	0	0	0
ThomasAM_2018b	3	0	2	0
VogtmannE_2016	0	0	1	0
WirbelJ_2018	37	22	25	16
YachidaS_2019	28	15	20	12
YeZ_2018	1	0	0	0
YuJ_2015	13	9	11	7
ZellerG_2014	3	2	2	2

In light of these results and more importantly given the lack of understanding in the field about which is the proper method that should be used (at least to the best of our knowledge), we prefer to i) keep the analysis in the manuscript as it is and ii) modify the text as follows (line 196):

We used Mann-Whitney U test to identify the set of significant taxa when relative abundance profiles were involved, while we adopted Fisher exact test to deal with presence/absence data. Although it is out of the scope of the present study to perform a comprehensive evaluation of available statistical tests, further investigation taking into account alternatives including methodologies that can deal with compositional issues [53,54] is warranted.

[53] Morton JT, Marotz C, Washburne A, Silverman J, Zaramela LS, Edlund A, et al. Establishing microbial composition measurement standards with reference frames. Nat Commun. 2019;10: 2719.

[54] Ling W, Zhao N, Plantinga AM, Launer LJ, Fodor AA, Meyer KA, et al. Powerful and robust non-parametric association testing for microbiome data via a zero-inflated quantile approach (ZINQ). Microbiome. 2021;9: 181.

Beyond these issues, I would recommend that the authors rethink the title of their paper, as it stands the classification performance is shown to be comparable in between both cases. The title makes an implication about qualitative profiles outperforming quantitative profiles. Furthermore the use of the term "metagenome" in the title is misleading since amplicon sequencing data is also presented in this paper.

Response

We modified the title as follows (line 1): “Host phenotype classification from human microbiome data is mainly driven by the presence of microbial taxa”.

Reviewer #3

This paper proposed a meta-analysis on 25 publicly available dataset of metagenomic studies and 30 public dataset of 16S rRNA studies, the major investigation purpose is whether presence/absence data could be a valid and efficient indicator for classifying samples. The conclusion is that by degrading abundance data to binary(presence/absence) data, the classification performance could be maintained without decreasing AUC in many parameter settings. This is an interesting paper for host-phenotype classification by only considering binary(presence/absence) data instead of abundance data, because it may suggest that it may be possible for designing microbe-based diagnostic tasks in future by the detection of the presence of a microbial taxa set rather than the complex abundance estimation by sequencing technology. The writing of the paper is very clear, however there are several concerns may be useful to consider, at least valuable to discuss:

Response
We thank the Reviewer for the overall appreciation of our work.

(1) The paper used Random forest, SVM, Lasso and Elastic Net for comparison, it is possible to adopt some updated classification approaches, for example:
https://pubmed.ncbi.nlm.nih.gov/32396115/
https://pubmed.ncbi.nlm.nih.gov/32657370/

Response
We thank the Reviewer for raising this point. The main goal of our study is to test differences between relative abundance and presence/absence profiles on classification results, and focusing more on a comparison among feature types rather than classification methods. Our analysis shows consistent outcomes across five (traditional) classification algorithms suggesting that findings are quite robust to the classifier choice. Anyhow, we have tried to performed additional analyses as follows:

- We tested five other classifiers available in the Python scikit-learn library (which is the library already used in our framework for the classification task). Our main messages were confirmed also by these other classification algorithms. At the same time, we didn't have consistent improvements of accuracy with respect to what was already reported in the manuscript. So we prefer to not add these additional classifiers in the manuscript since they do not add too much to the story;
- The source code for the first manuscript suggested by the Reviewer is not available. Anyhow, authors already reported in this original publication that the proposed algorithm didn't outperform other methods such as random forest which is already considered in our analysis. We were not able to properly install and run the software associated with the second paper.

In conclusion, we think that doing an extensive evaluation of *all* available classifiers is out of the scope of the present study. At the same time, we feel that testing other classification approaches, including the ones specifically proposed for the analysis of metagenomic data as suggested by the Reviewer, is something relevant for future studies in the microbiome field. We added a couple of sentences about this aspect in the text (line 387):

Moreover, although doing an extensive evaluation of existing classifiers is out of the scope of the present study, maximization of classification accuracies may be reached by adopting other

classification approaches including the ones specifically proposed for microbiome data analysis [61,62].

[61] Reiman D, Metwally AA, Sun J, Dai Y. PopPhy-CNN: A Phylogenetic Tree Embedded Architecture for Convolutional Neural Networks to Predict Host Phenotype From Metagenomic Data. IEEE J Biomed Health Inform. 2020;24: 2993–3001.
[62] Rahman MA, Rangwala H. IDMIL: an alignment-free Interpretable Deep Multiple Instance Learning (MIL) for predicting disease from whole-metagenomic data. Bioinformatics. 2020;36: i39–i47.

(2) The paper designed many experiments by using a lot of datasets, it is useful to provide a website link for downloading the processed dataset for comparison and reproduce the results.

Response
As suggested also by Reviewer 2, we created a public repository hosted in GitHub (https://github.com/RGilib/giliberti-meta-analysis-2022) and more specifically a detailed Wiki section (https://github.com/RGilib/giliberti-meta-analysis-2022/wiki). Here, we provide a tutorial aiming at going through the entire pipeline in addition to the repository with all the data and code necessary to replicate all results and figures/tables of the paper.
We added this sentence in the Data availability statement:
The data and source code used to produce the results and analyses presented in this manuscript are available on a GitHub repository at https://github.com/RGilib/giliberti-meta-analysis-2022.

(3)Traditionally, classification methods including Random forest, SVM, Lasso and Elastic Net are designed for continuous data instead of binary data (presence/absence data). When the data form is changed, the algorithm may not adapted to it. The author may explain why these methods could work on both continuous data (abundance data) and binary data (presence/absence data)

Response
This is an interesting point. We agree with the comment formulated by the Reviewer, which is actually reinforcing our message. We show that classification accuracies are not affected when relative abundance features are degraded to presence/absence profiles. This happens using classification methods that are "optimal" for continuous data and potentially "suboptimal" for binary data, therefore accuracies may be even better when models on presence/absence profiles are trained using classifiers more designed for binary data. We added this consideration in the text as follows (line 382):
Results were robust to the choice of the classifier. This was obtained by considering different traditional classification algorithms that are designed for continuous data and potentially "suboptimal" when applied on binary data. This actually reinforces our findings, meaning that accuracies may be even better when models on presence/absence profiles are trained using classifiers more designed for binary data.

Have the authors made all data and (if applicable) computational code underlying the findings in their manuscript fully available?
The PLOS Data policy requires authors to make all data and code underlying the findings described in their manuscript fully available without restriction, with rare exception (please refer to the Data Availability Statement in the manuscript PDF file). The data and code should be provided as part of the manuscript or its supporting information, or deposited to a public repository. For example, in addition to summary statistics, the data points behind means, medians and variance measures should be available. If there are restrictions on publicly sharing data or code —e.g. participant privacy or use of data from a third party—those must be specified.

Reviewer #1: Yes

Reviewer #2: No:

Reviewer #3: Yes

Response
We fixed this issue by creating a public repository hosted in GitHub (https://github.com/RGilib/giliberti-meta-analysis-2022). Here we provide a Wiki section (https://github.com/RGilib/giliberti-meta-analysis-2022/wiki) with all the data and code necessary to replicate results and figures/tables of the paper.
We added this sentence in the Data availability statement:
The data and source code used to produce the results and analyses presented in this manuscript are available on a GitHub repository at https://github.com/RGilib/giliberti-meta-analysis-2022.
