# Supplementary material for: Host phenotype classification from human microbiome data is mainly driven by the presence of microbial taxa
Source: PLoS Comput Biol. 2022 Apr 21;18(4):e1010066. doi: 10.1371/journal.pcbi.1010066 (PMC9064115; doi:10.1371/journal.pcbi.1010066)
Supplement: Attachment — Submitted filename: Presence absence meta analysis - Rebuttal letter.pdf [file pcbi.1010066.s025.pdf]

## Editors

**We consider that the scientific questions have been addressed in this revision. However, before accepting the manuscript, we ask that the authors revise the figures, particularly the supplemental ones, for improved readability and accessibility.**

<u>Response</u>

We thank you again for overall positive feedback about our work. We revised all figures, including supplemental ones, for improved readability and accessibility.

**The fonts and markers are often too small to read comfortably even though there appears to be space available. This is particularly true in the supplemental figures, but some of the main figures could also be improved.**

<u>Response</u>

We enlarged fonts and markers in all figures, including supplemental ones, to guarantee a more comfortable reading.

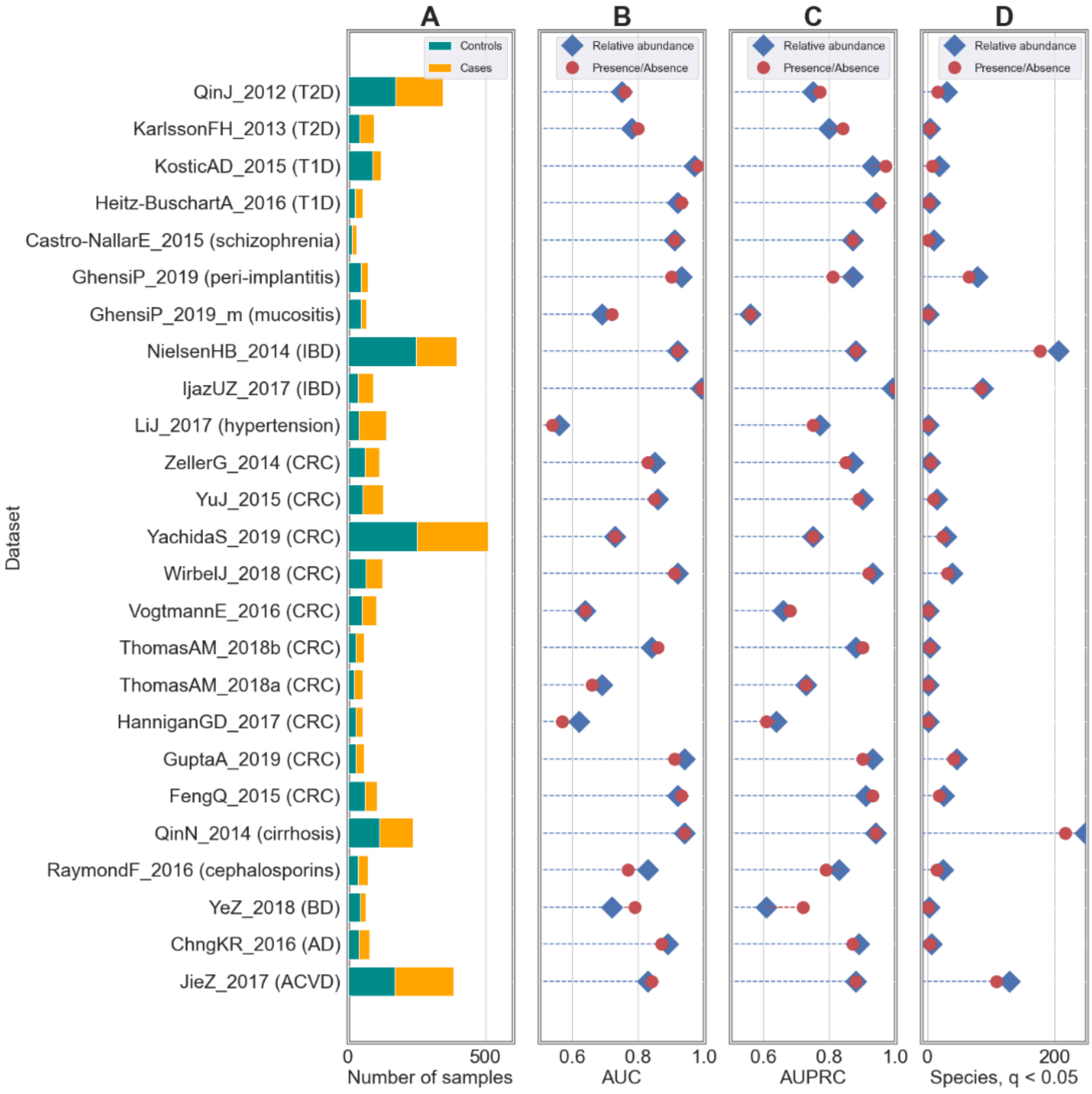


**Fig. 1.**

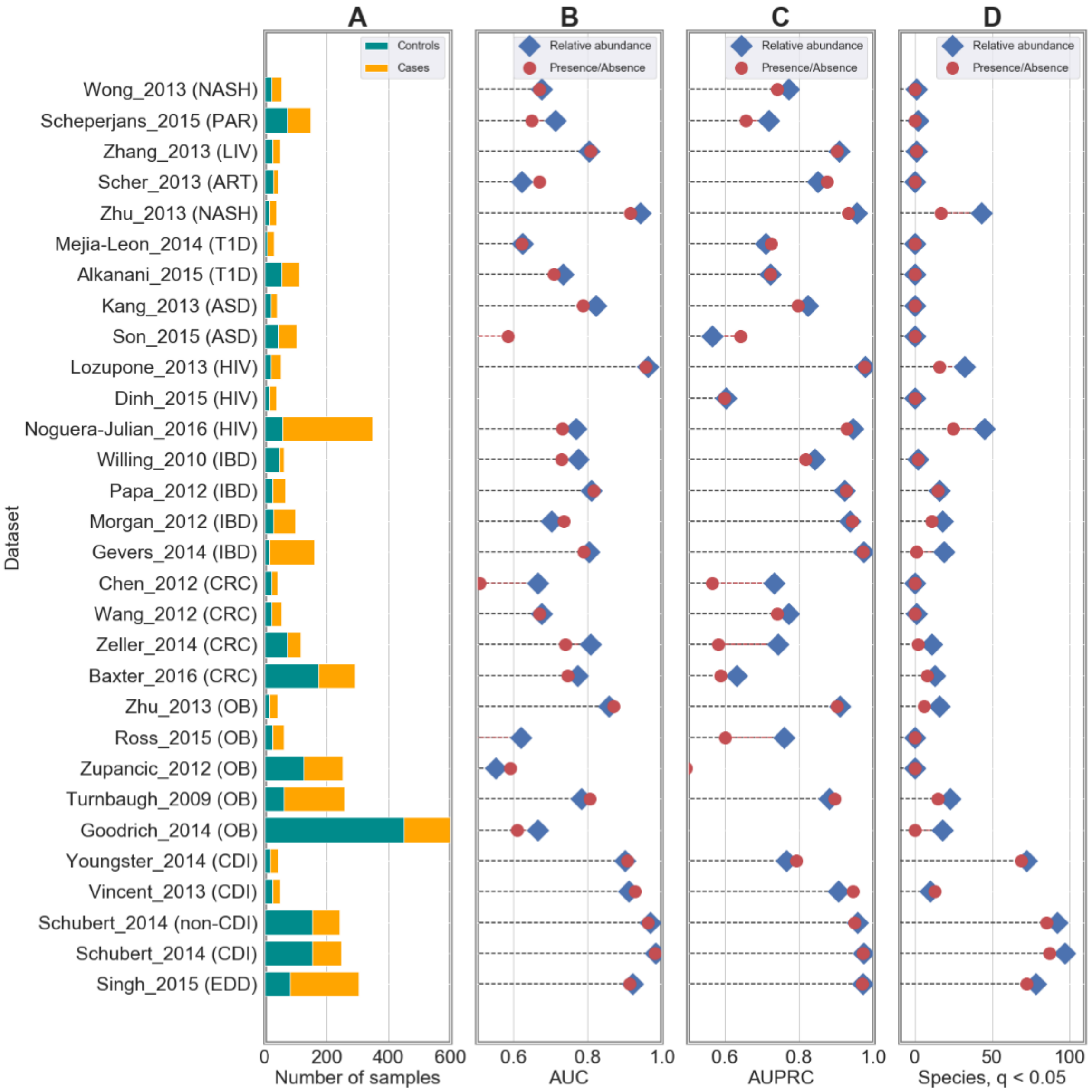
A
B
C
D
Controls
Cases
Relative abundance
Presence/Absence
Dataset
Wong_2013 (NASH)
Scheperjans_2015 (PAR)
Zhang_2013 (LIV)
Scher_2013 (ART)
Zhu_2013 (NASH)
Mejia-Leon_2014 (T1D)
Alkanani_2015 (T1D)
Kang_2013 (ASD)
Son_2015 (ASD)
Lozupone_2013 (HIV)
Dinh_2015 (HIV)
Noguera-Julian_2016 (HIV)
Willing_2010 (IBD)
Papa_2012 (IBD)
Morgan_2012 (IBD)
Gevers_2014 (IBD)
Chen_2012 (CRC)
Wang_2012 (CRC)
Zeller_2014 (CRC)
Baxter_2016 (CRC)
Zhu_2013 (OB)
Ross_2015 (OB)
Zupancic_2012 (OB)
Turnbaugh_2009 (OB)
Goodrich_2014 (OB)
Youngster_2014 (CDI)
Vincent_2013 (CDI)
Schubert_2014 (non-CDI)
Schubert_2014 (CDI)
Singh_2015 (EDD)
0
200
400
600
Number of samples
0.6
0.8
1.0
AUC
0.6
0.8
1.0
AUPRC
0
50
100
Species, q < 0.05

**Fig. 2.**

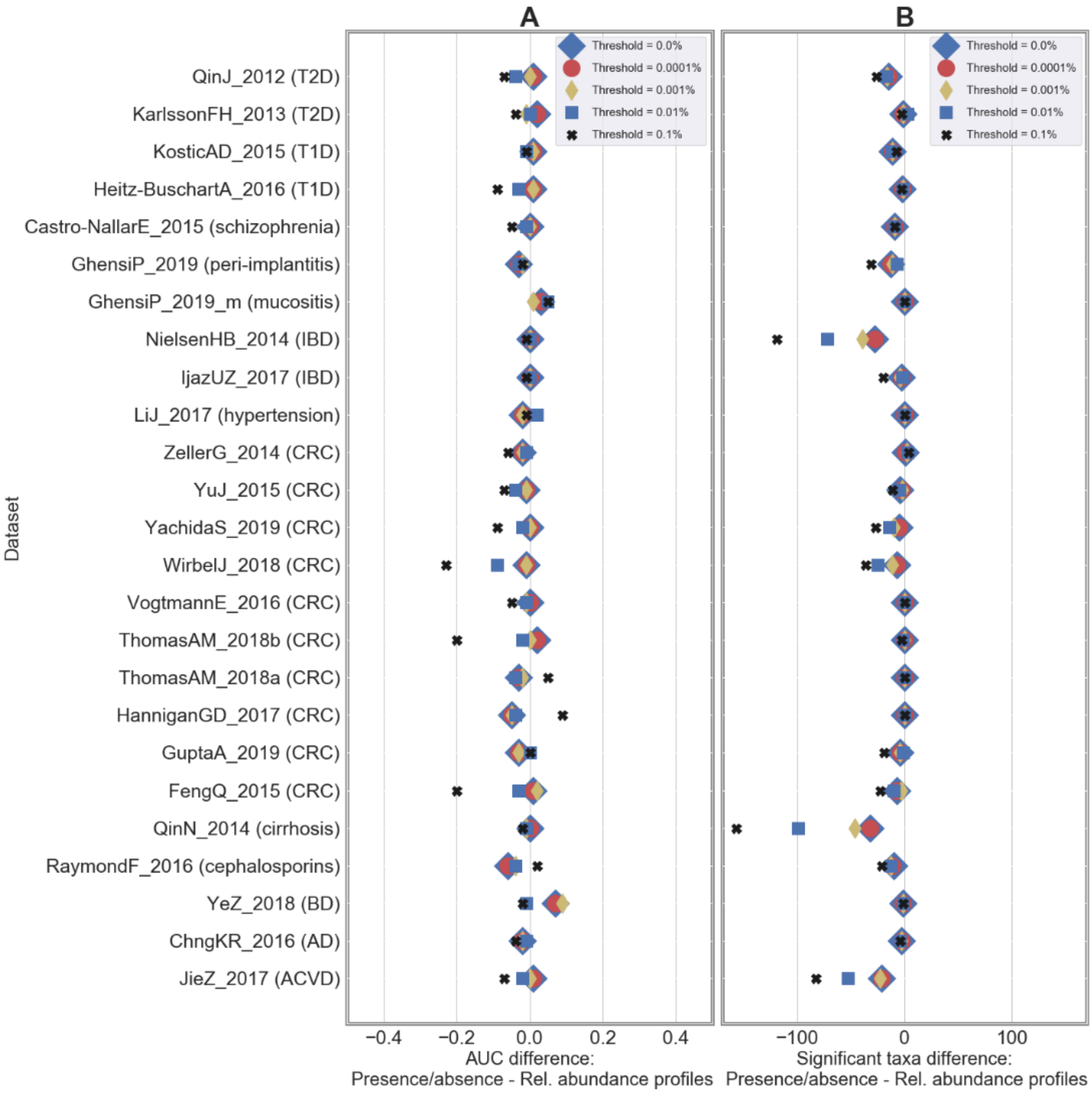
A
B
Threshold = 0.0%
Threshold = 0.0001%
Threshold = 0.001%
Threshold = 0.01%
Threshold = 0.1%
QinJ_2012 (T2D)
KarlssonFH_2013 (T2D)
KosticAD_2015 (T1D)
Heitz-BuschartA_2016 (T1D)
Castro-NallarE_2015 (schizophrenia)
GhensiP_2019 (peri-implantitis)
GhensiP_2019_m (mucositis)
NielsenHB_2014 (IBD)
IjazUZ_2017 (IBD)
LiJ_2017 (hypertension)
ZellerG_2014 (CRC)
YuJ_2015 (CRC)
YachidaS_2019 (CRC)
WirbelJ_2018 (CRC)
VogtmannE_2016 (CRC)
ThomasAM_2018b (CRC)
ThomasAM_2018a (CRC)
HanniganGD_2017 (CRC)
GuptaA_2019 (CRC)
FengQ_2015 (CRC)
QinN_2014 (cirrhosis)
RaymondF_2016 (cephalosporins)
YeZ_2018 (BD)
ChngKR_2016 (AD)
JieZ_2017 (ACVD)
Dataset
−0.4 −0.2 0.0 0.2 0.4
AUC difference:
Presence/absence - Rel. abundance profiles
−100 0 100
Significant taxa difference:
Presence/absence - Rel. abundance profiles

**Fig. 3.**

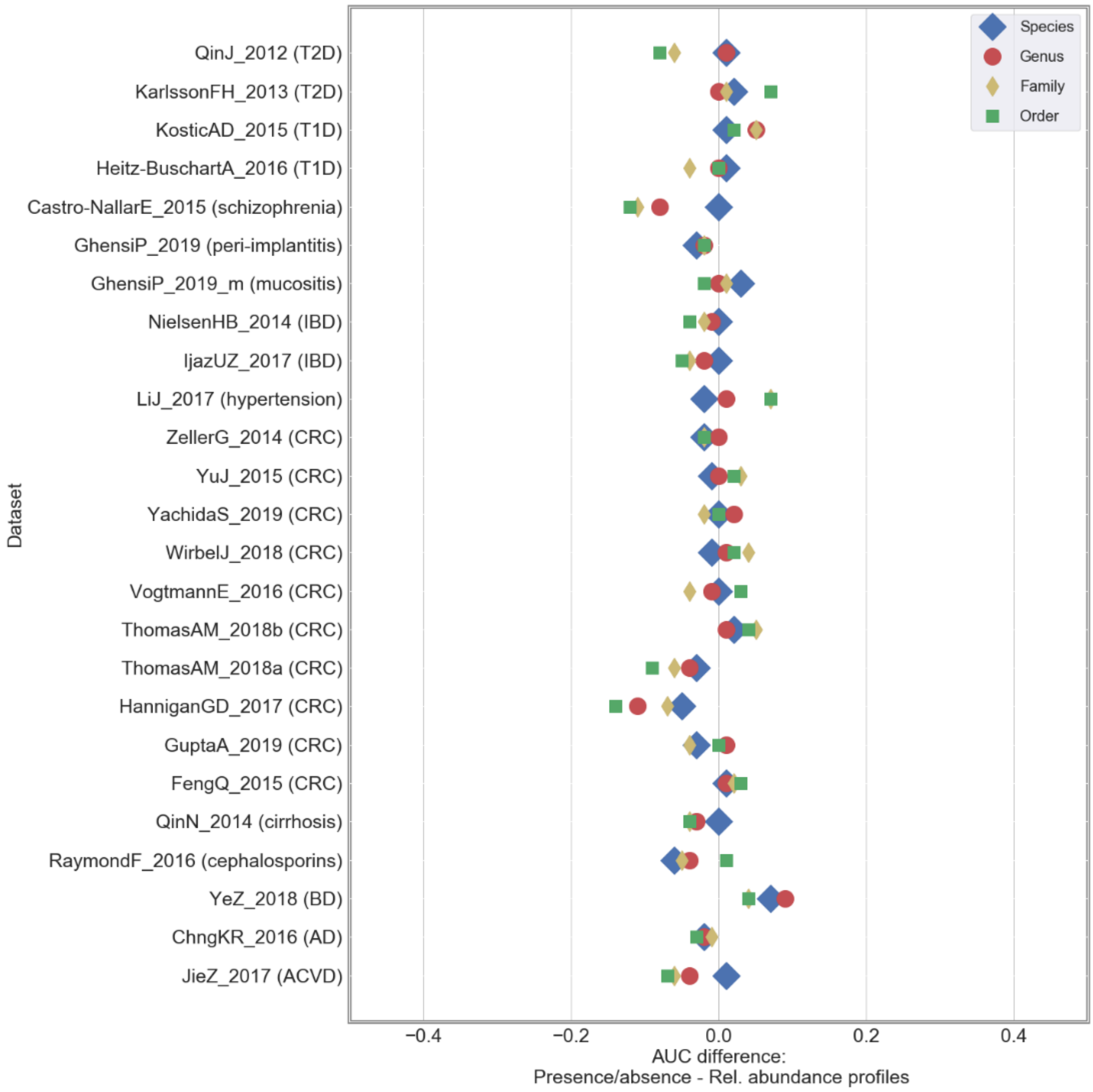
QinJ_2012 (T2D)
KarlssonFH_2013 (T2D)
KosticAD_2015 (T1D)
Heitz-BuschartA_2016 (T1D)
Castro-NallarE_2015 (schizophrenia)
GhensiP_2019 (peri-implantitis)
GhensiP_2019_m (mucositis)
NielsenHB_2014 (IBD)
IjazUZ_2017 (IBD)
LiJ_2017 (hypertension)
ZellerG_2014 (CRC)
YuJ_2015 (CRC)
YachidaS_2019 (CRC)
WirbelJ_2018 (CRC)
VogtmannE_2016 (CRC)
ThomasAM_2018b (CRC)
ThomasAM_2018a (CRC)
HanniganGD_2017 (CRC)
GuptaA_2019 (CRC)
FengQ_2015 (CRC)
QinN_2014 (cirrhosis)
RaymondF_2016 (cephalosporins)
YeZ_2018 (BD)
ChngKR_2016 (AD)
JieZ_2017 (ACVD)
Dataset
−0.4
−0.2
0.0
0.2
0.4
AUC difference:
Presence/absence - Rel. abundance profiles
Species
Genus
Family
Order

**Fig. 4.**

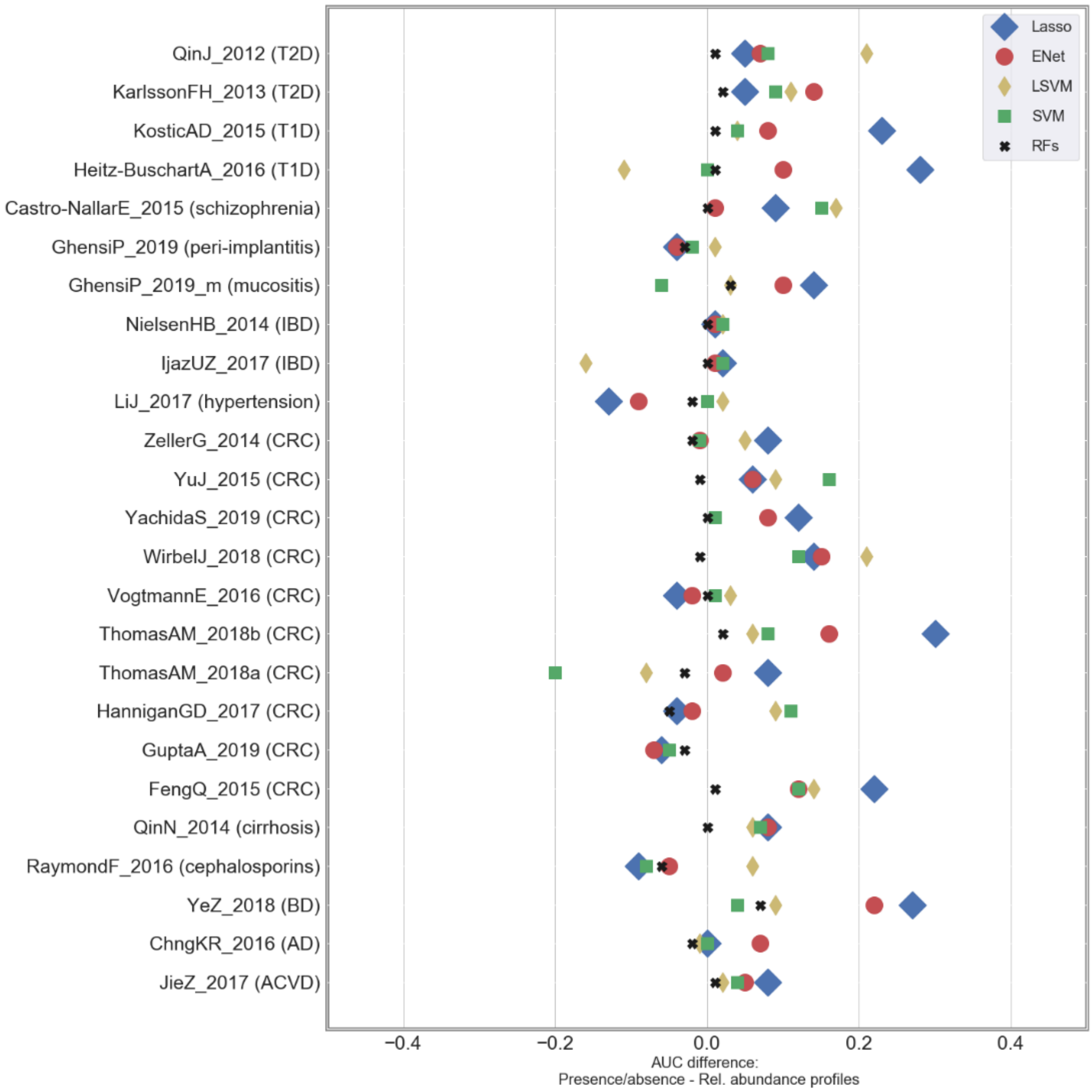

QinJ_2012 (T2D)
KarlssonFH_2013 (T2D)
KosticAD_2015 (T1D)
Heitz-BuschartA_2016 (T1D)
Castro-NallarE_2015 (schizophrenia)
GhensiP_2019 (peri-implantitis)
GhensiP_2019_m (mucositis)
NielsenHB_2014 (IBD)
IjazUZ_2017 (IBD)
LiJ_2017 (hypertension)
ZellerG_2014 (CRC)
YuJ_2015 (CRC)
YachidaS_2019 (CRC)
WirbelJ_2018 (CRC)
VogtmannE_2016 (CRC)
ThomasAM_2018b (CRC)
ThomasAM_2018a (CRC)
HanniganGD_2017 (CRC)
GuptaA_2019 (CRC)
FengQ_2015 (CRC)
QinN_2014 (cirrhosis)
RaymondF_2016 (cephalosporins)
YeZ_2018 (BD)
ChngKR_2016 (AD)
JieZ_2017 (ACVD)
Lasso
ENet
LSVM
SVM
RFs
−0.4
−0.2
0.0
0.2
0.4
AUC difference:
Presence/absence - Rel. abundance profiles


**Fig. 5.**

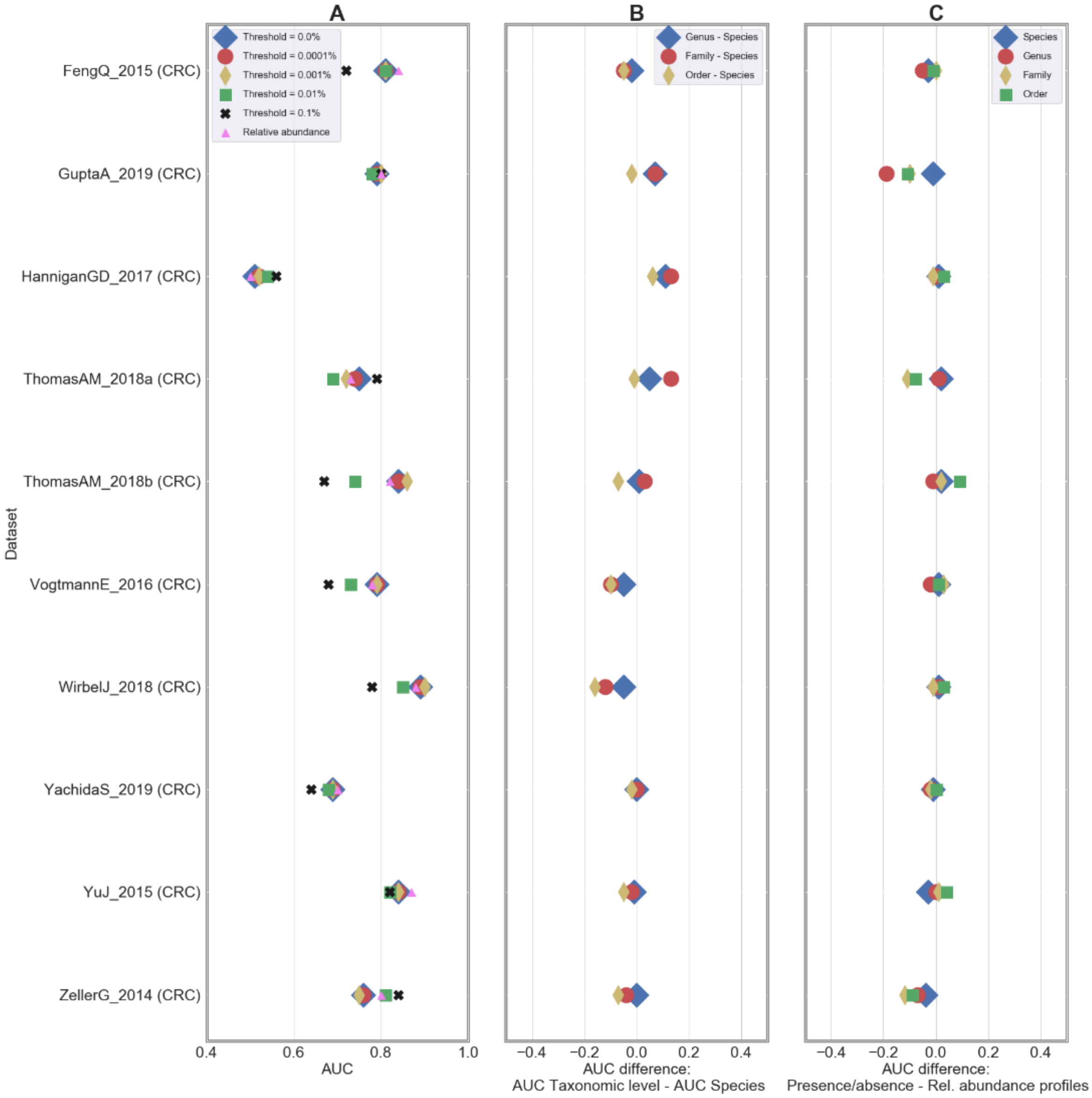
A
B
C
Threshold = 0.0%
Threshold = 0.0001%
Threshold = 0.001%
Threshold = 0.01%
Threshold = 0.1%
Relative abundance
Genus - Species
Family - Species
Order - Species
Species
Genus
Family
Order
FengQ_2015 (CRC)
GuptaA_2019 (CRC)
HanniganGD_2017 (CRC)
ThomasAM_2018a (CRC)
ThomasAM_2018b (CRC)
VogtmannE_2016 (CRC)
WirbelJ_2018 (CRC)
YachidaS_2019 (CRC)
YuJ_2015 (CRC)
ZellerG_2014 (CRC)
Dataset
0.4
0.6
0.8
1.0
AUC
−0.4
−0.2
0.0
0.2
0.4
AUC difference:
AUC Taxonomic level - AUC Species
AUC difference:
Presence/absence - Rel. abundance profiles

**Fig. 6.**

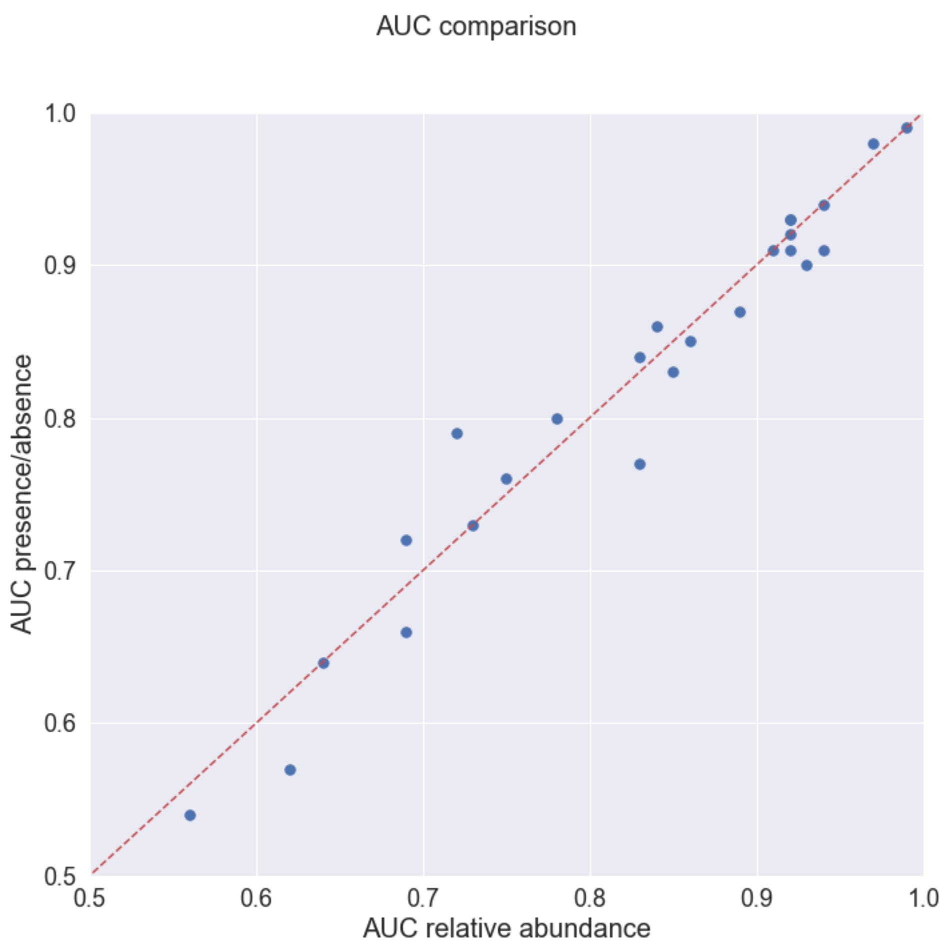

AUC comparison
1.0
0.9
0.8
0.7
0.6
0.5
AUC presence/absence
0.5
0.6
0.7
0.8
0.9
1.0
AUC relative abundance


**S1 Fig.**

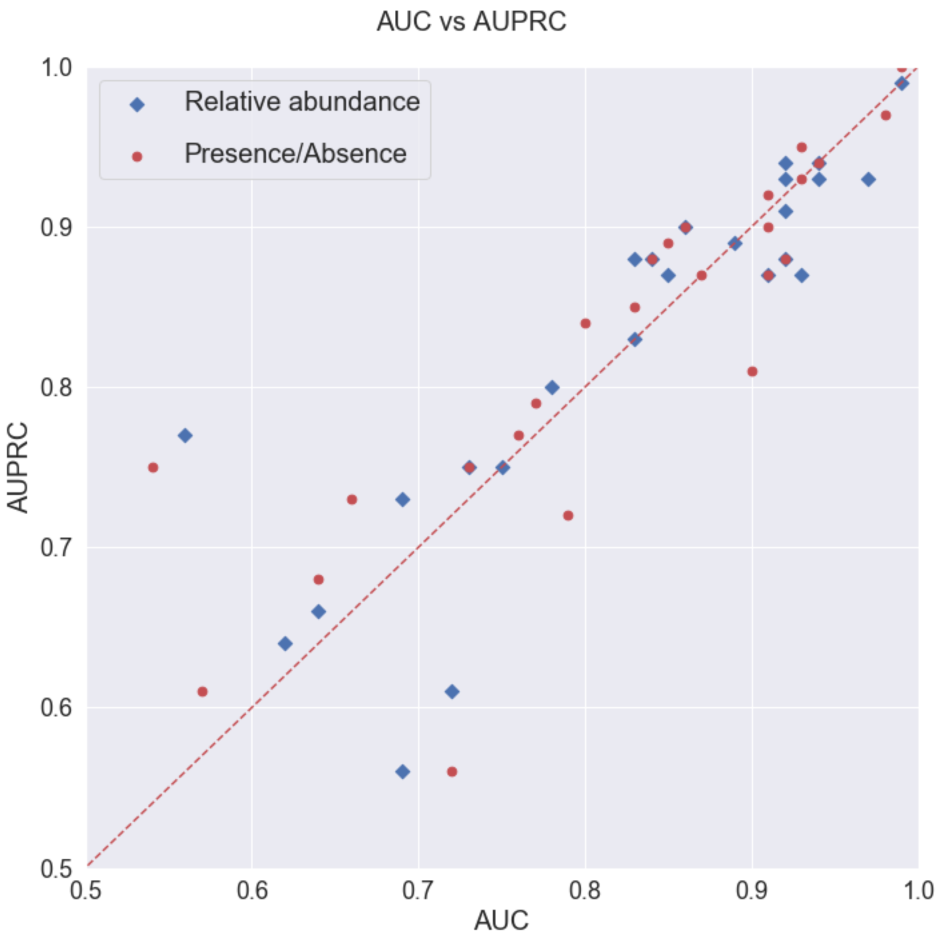

AUC vs AUPRC
Relative abundance
Presence/Absence
1.0
0.9
0.8
0.7
0.6
0.5
AUPRC
0.5
0.6
0.7
0.8
0.9
1.0
AUC


**S2 Fig.**

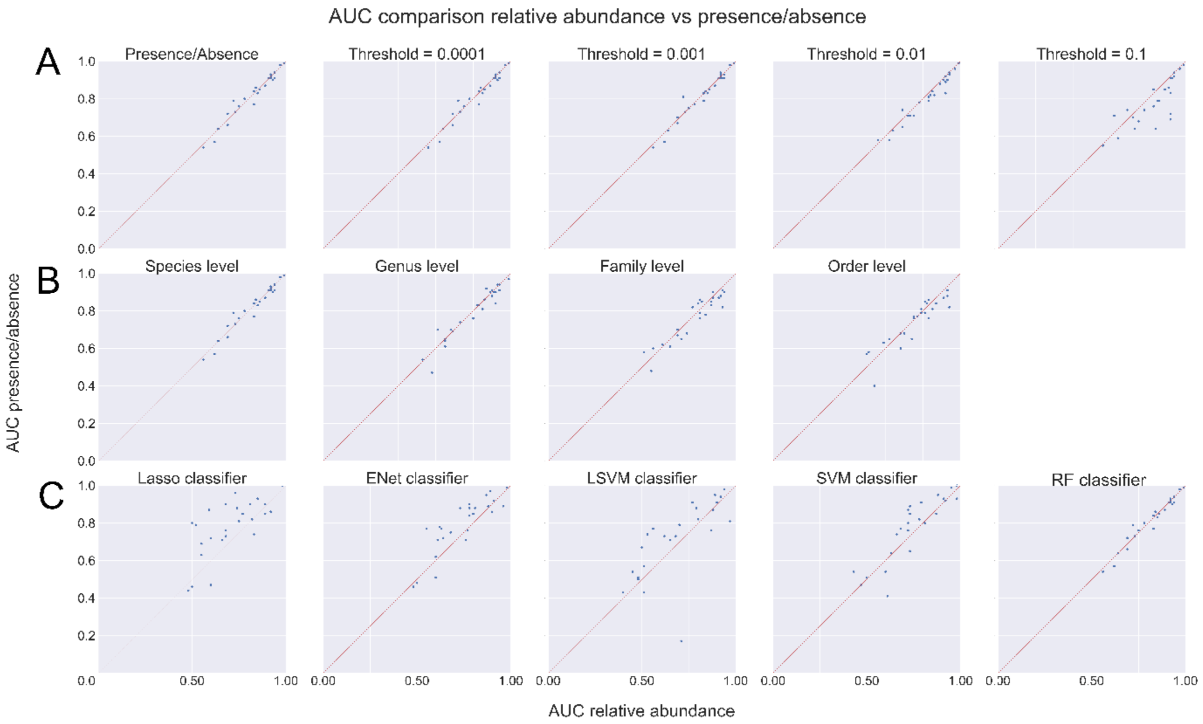
AUC comparison relative abundance vs presence/absence
A
Presence/Absence
Threshold = 0.0001
Threshold = 0.001
Threshold = 0.01
Threshold = 0.1
B
Species level
Genus level
Family level
Order level
C
Lasso classifier
ENet classifier
LSVM classifier
SVM classifier
RF classifier
AUC presence/absence
AUC relative abundance
0.0
0.2
0.4
0.6
0.8
1.0
0.00
0.50
1.00

**S3 Fig.**

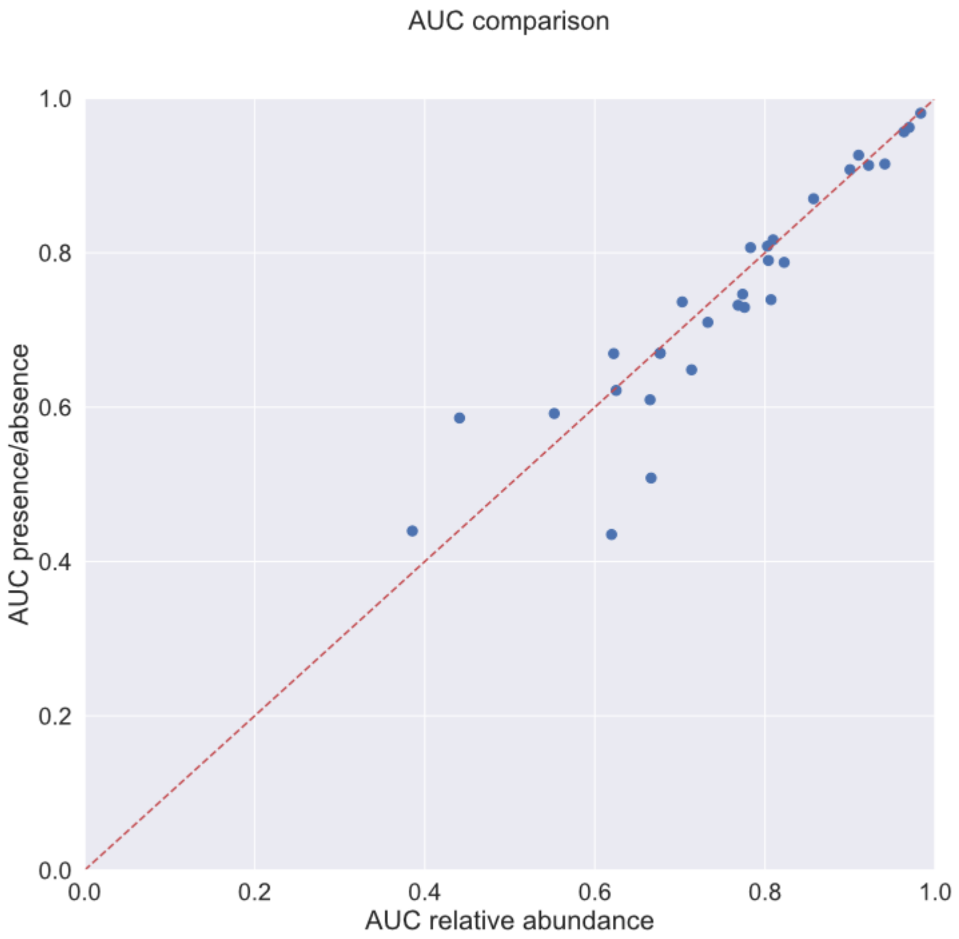
AUC comparison
AUC presence/absence
AUC relative abundance
0.0
0.2
0.4
0.6
0.8
1.0

**S4 Fig.**

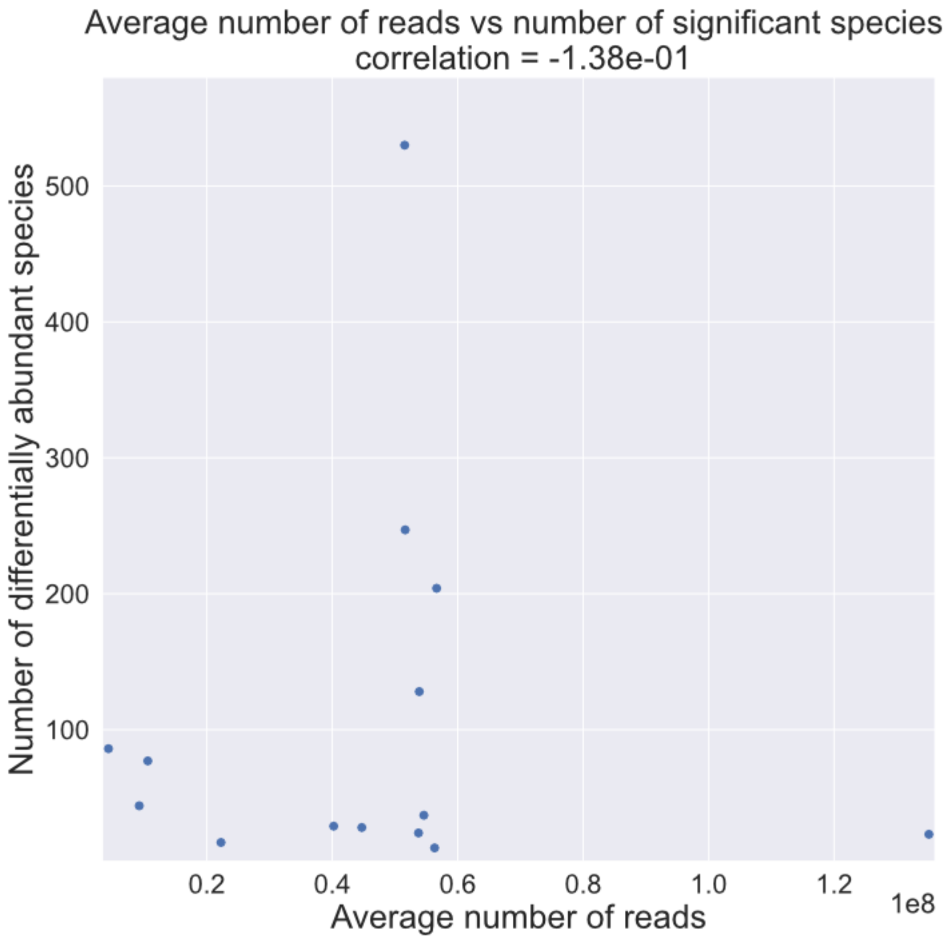

Average number of reads vs number of significant species
correlation = -1.38e-01
Number of differentially abundant species
500
400
300
200
100
0.2
0.4
0.6
0.8
1.0
1.2
1e8
Average number of reads


**S5 Fig.**

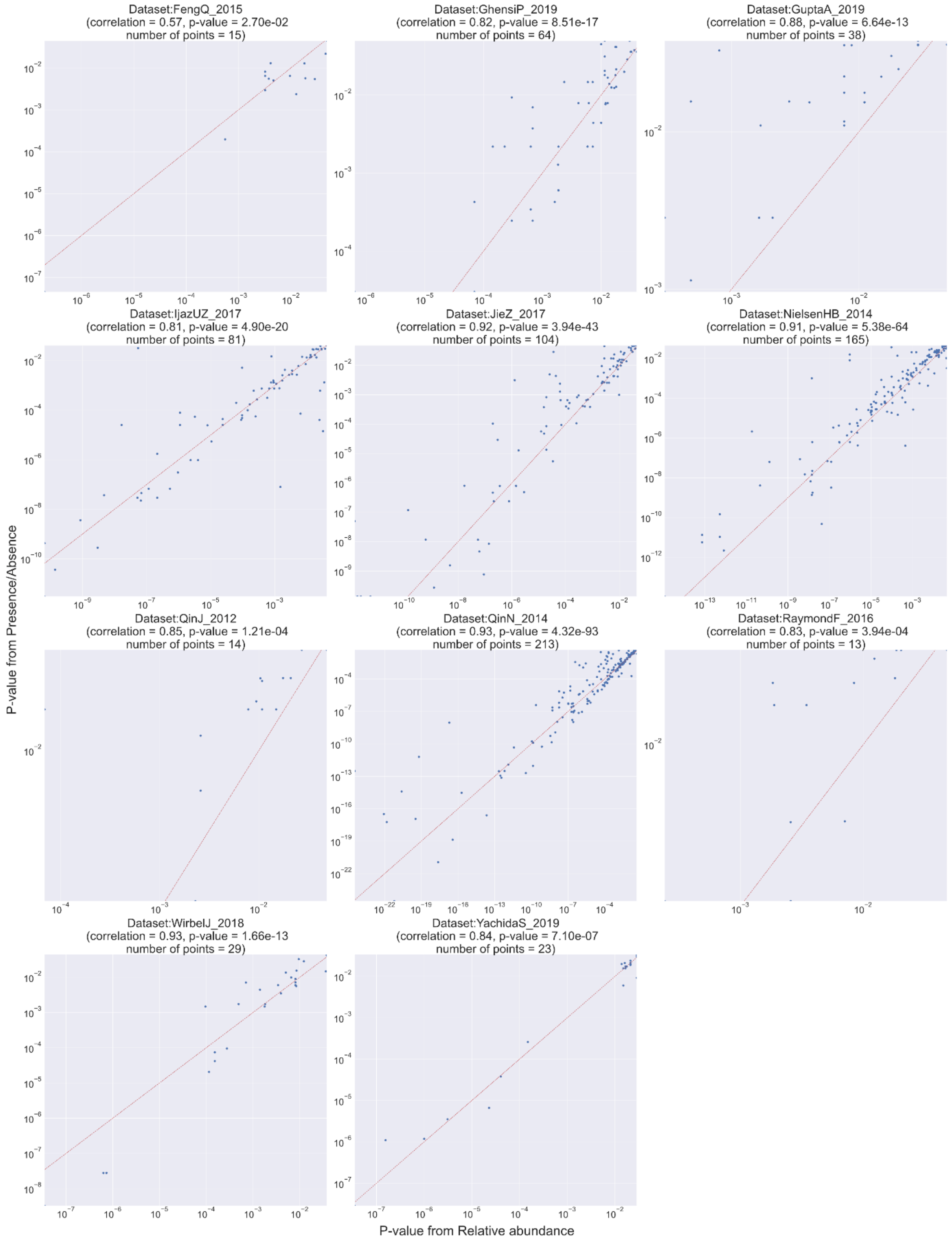
Dataset:FengQ_2015
(correlation = 0.57, p-value = 2.70e-02
number of points = 15)
Dataset:GhensiP_2019
(correlation = 0.82, p-value = 8.51e-17
number of points = 64)
Dataset:GuptaA_2019
(correlation = 0.88, p-value = 6.64e-13
number of points = 38)
Dataset:IjazUZ_2017
(correlation = 0.81, p-value = 4.90e-20
number of points = 81)
Dataset:JieZ_2017
(correlation = 0.92, p-value = 3.94e-43
number of points = 104)
Dataset:NielsenHB_2014
(correlation = 0.91, p-value = 5.38e-64
number of points = 165)
Dataset:QinJ_2012
(correlation = 0.85, p-value = 1.21e-04
number of points = 14)
Dataset:QinN_2014
(correlation = 0.93, p-value = 4.32e-93
number of points = 213)
Dataset:RaymondF_2016
(correlation = 0.83, p-value = 3.94e-04
number of points = 13)
Dataset:WirbelJ_2018
(correlation = 0.93, p-value = 1.66e-13
number of points = 29)
Dataset:YachidaS_2019
(correlation = 0.84, p-value = 7.10e-07
number of points = 23)
P-value from Presence/Absence
P-value from Relative abundance

**S6 Fig.**

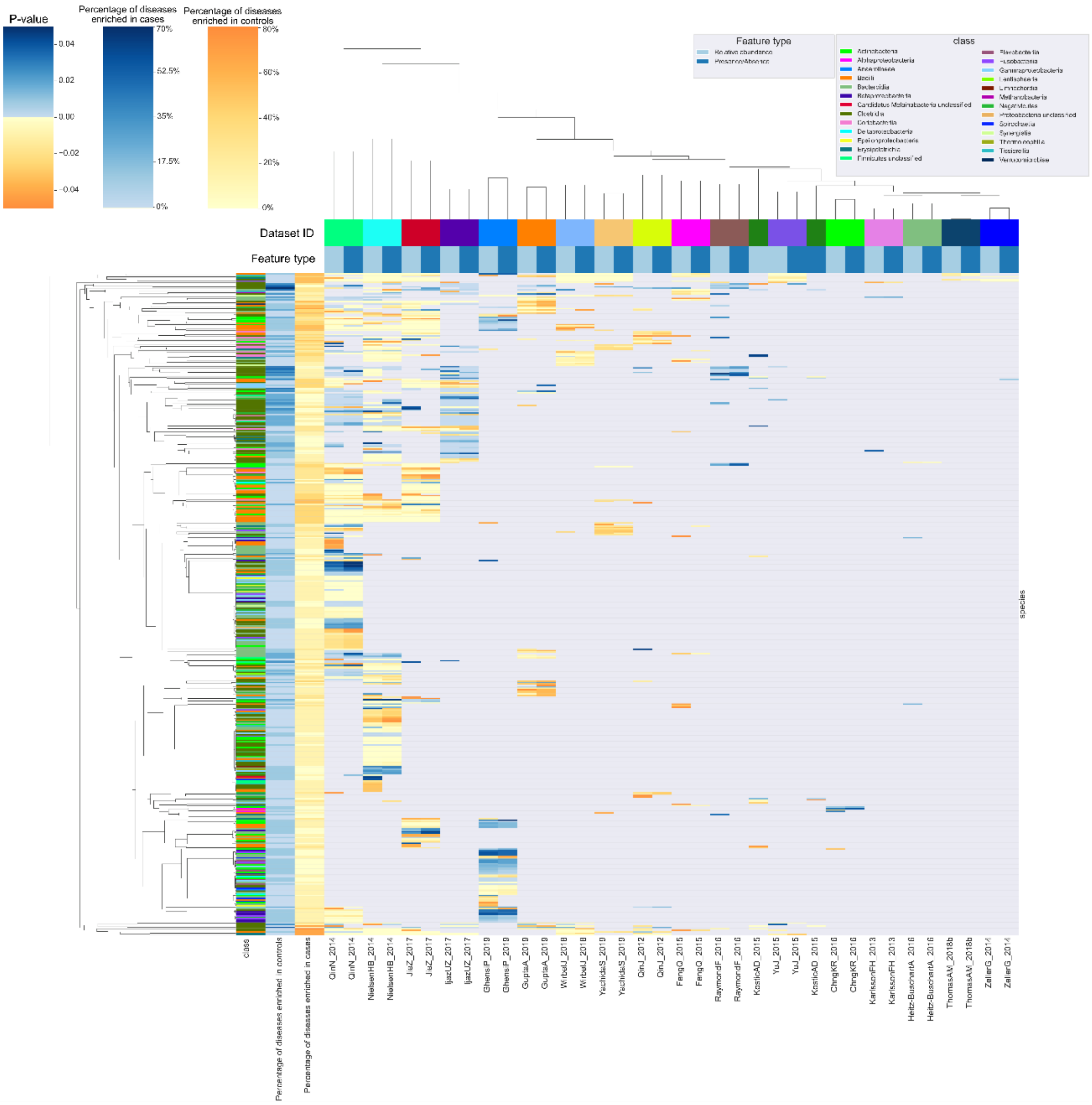
P-value
Percentage of diseases enriched in cases
Percentage of diseases enriched in controls
Feature type
Relative abundance
Presence/Absence
class
Actinobacteria
Alphaproteobacteria
Bacilli
Bacteroidia
Betaproteobacteria
Candidatus Melainabacteria unclassified
Clostridia
Coriobacteriia
Deltaproteobacteria
Epsilonproteobacteria
Erysipelotrichia
Firmicutes unclassified
Fusobacteriia
Gammaproteobacteria
Methanobacteria
Negativicutes
Proteobacteria unclassified
Spirochaetia
Synergistia
Tissierellia
Verrucomicrobiae
Dataset ID
Feature type
species
class
Percentage of diseases enriched in controls
Percentage of diseases enriched in cases
QinN_2014
NielsenHB_2014
JieZ_2017
IjazUZ_2017
GhensiP_2019
GuptaA_2019
WirbelJ_2018
YachidaS_2019
QinJ_2012
FengQ_2015
RaymondF_2016
KosticAD_2015
YuJ_2015
ChngKR_2016
KarlssonFH_2013
Heitz-BuschartA_2016
ThomasAM_2018b
ZellerG_2014

S7 Fig.

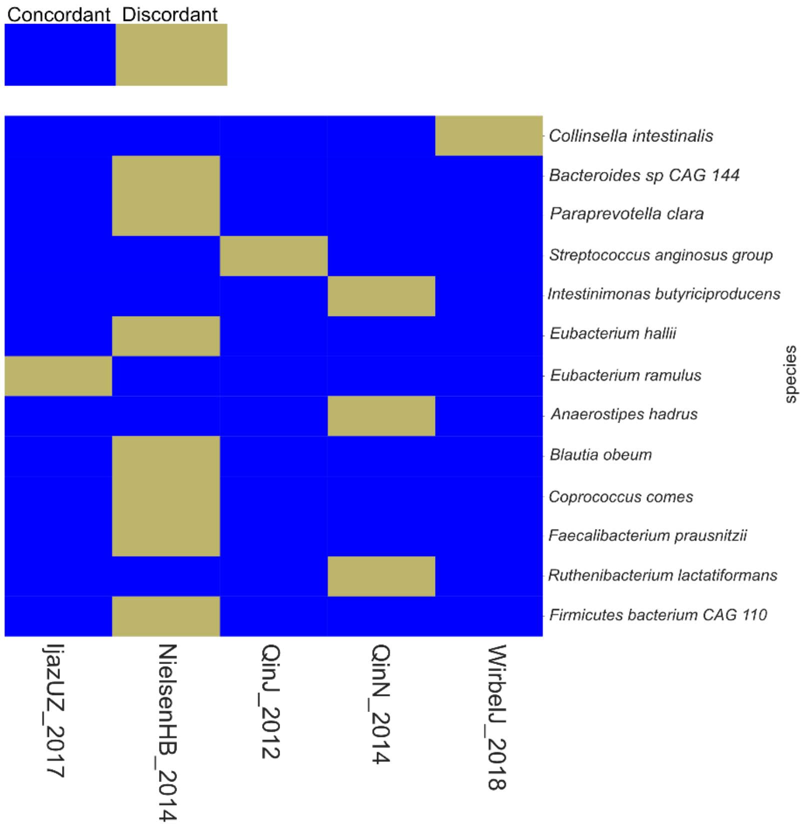
Concordant
Discordant
Collinsella intestinalis
Bacteroides sp CAG 144
Paraprevotella clara
Streptococcus anginosus group
Intestinimonas butyriciproducens
Eubacterium hallii
Eubacterium ramulus
Anaerostipes hadrus
Blautia obeum
Coprococcus comes
Faecalibacterium prausnitzii
Ruthenibacterium lactatiformans
Firmicutes bacterium CAG 110
species
IjazUZ_2017
NielsenHB_2014
QinJ_2012
QinN_2014
WirbelJ_2018

**S8 Fig.**

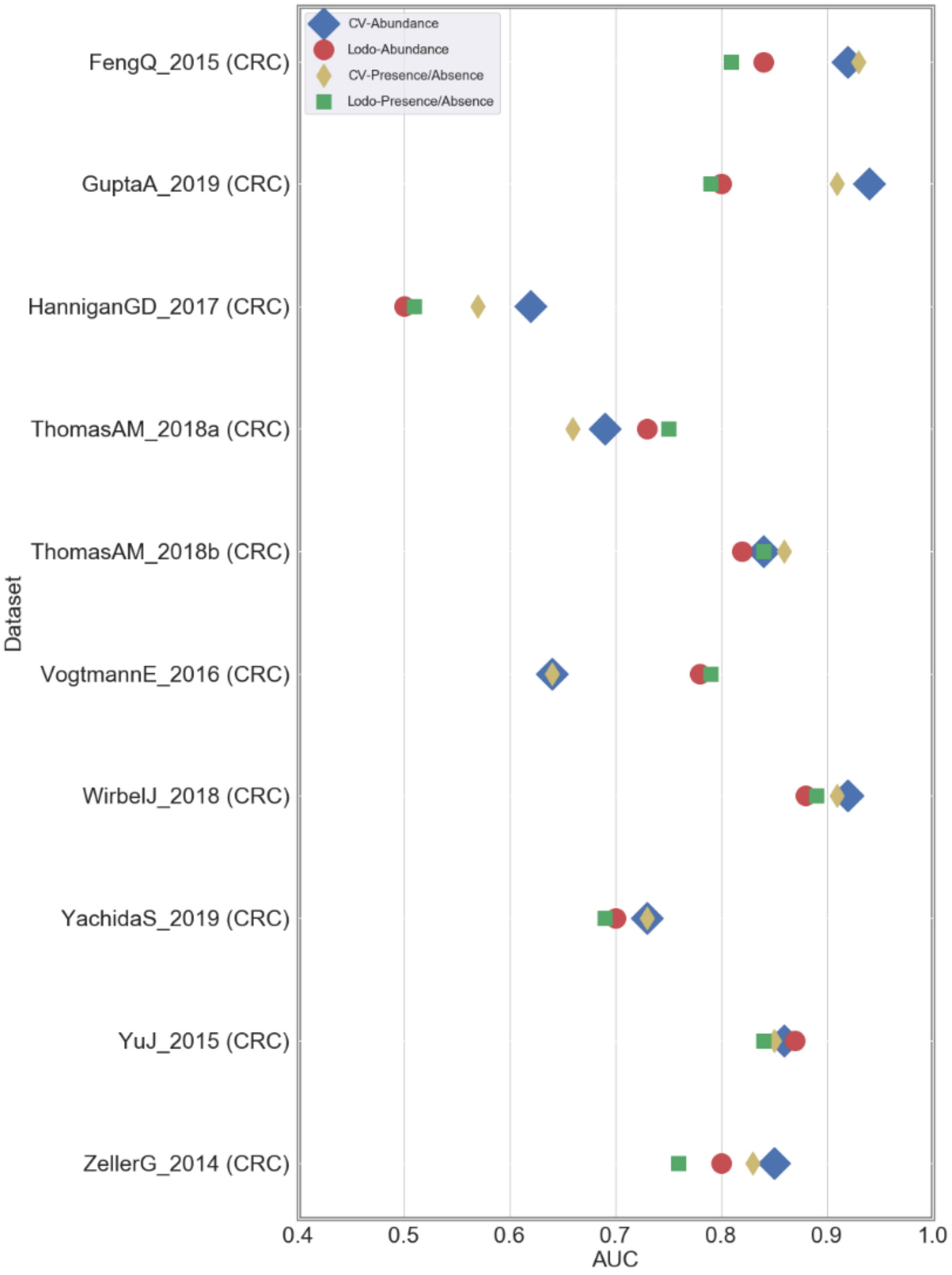

CV-Abundance
Lodo-Abundance
CV-Presence/Absence
Lodo-Presence/Absence
FengQ_2015 (CRC)
GuptaA_2019 (CRC)
HanniganGD_2017 (CRC)
ThomasAM_2018a (CRC)
ThomasAM_2018b (CRC)
VogtmannE_2016 (CRC)
WirbelJ_2018 (CRC)
YachidaS_2019 (CRC)
YuJ_2015 (CRC)
ZellerG_2014 (CRC)
Dataset
0.4
0.5
0.6
0.7
0.8
0.9
1.0
AUC


**S9 Fig.**

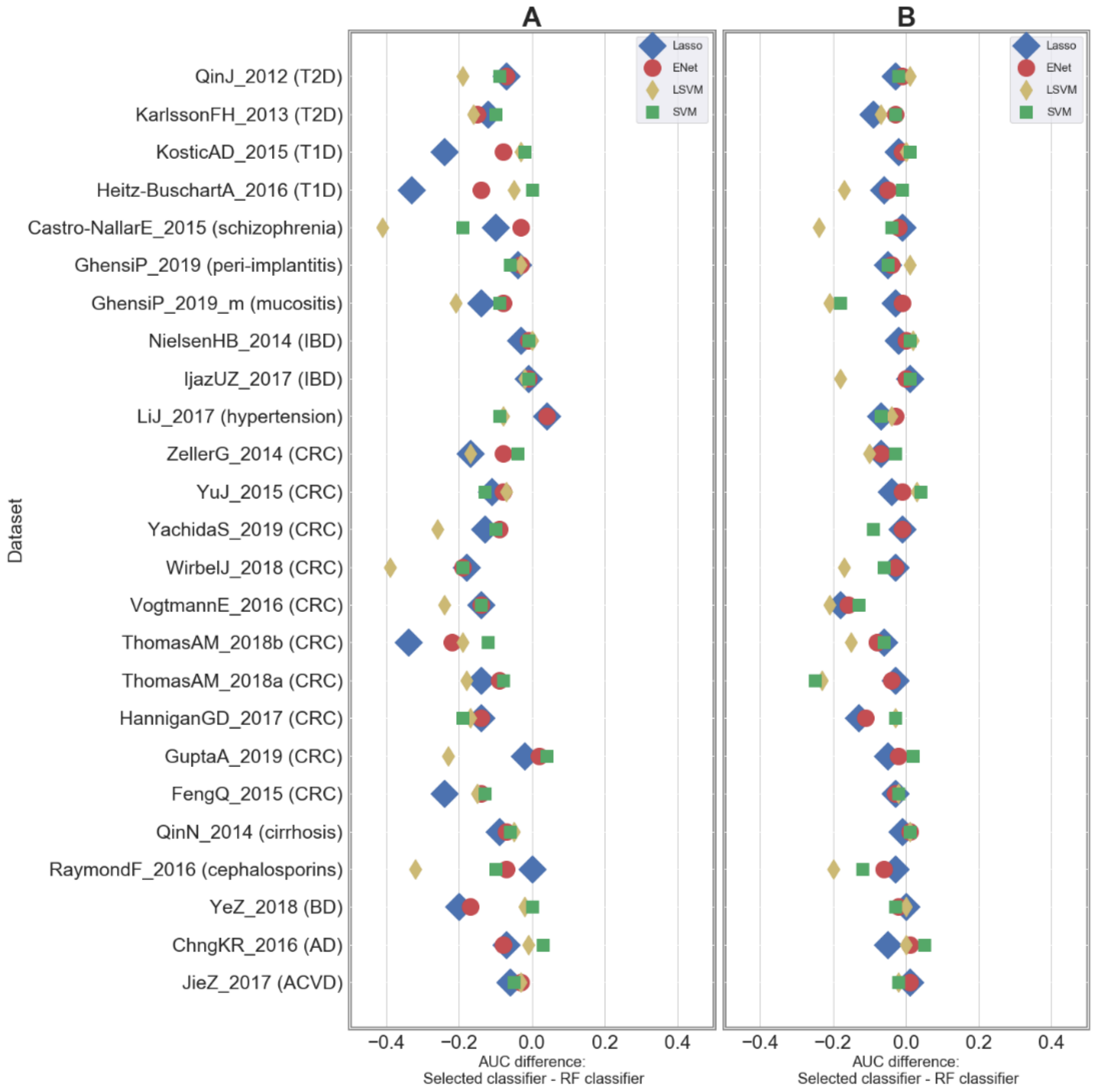
A
B
Lasso
ENet
LSVM
SVM
Dataset
QinJ_2012 (T2D)
KarlssonFH_2013 (T2D)
KosticAD_2015 (T1D)
Heitz-BuschartA_2016 (T1D)
Castro-NallarE_2015 (schizophrenia)
GhensiP_2019 (peri-implantitis)
GhensiP_2019_m (mucositis)
NielsenHB_2014 (IBD)
IjazUZ_2017 (IBD)
LiJ_2017 (hypertension)
ZellerG_2014 (CRC)
YuJ_2015 (CRC)
YachidaS_2019 (CRC)
WirbelJ_2018 (CRC)
VogtmannE_2016 (CRC)
ThomasAM_2018b (CRC)
ThomasAM_2018a (CRC)
HanniganGD_2017 (CRC)
GuptaA_2019 (CRC)
FengQ_2015 (CRC)
QinN_2014 (cirrhosis)
RaymondF_2016 (cephalosporins)
YeZ_2018 (BD)
ChngKR_2016 (AD)
JieZ_2017 (ACVD)
−0.4
−0.2
0.0
0.2
0.4
AUC difference:
Selected classifier - RF classifier

**S10 Fig.**

**We also ask that Figs S7 and S8 be redone using a coulorblind-safe color scheme rather than rely on red/green distinction.**

<u>Response</u>

We updated Figs S7 and S8 by using a colourblind-safe color scheme:

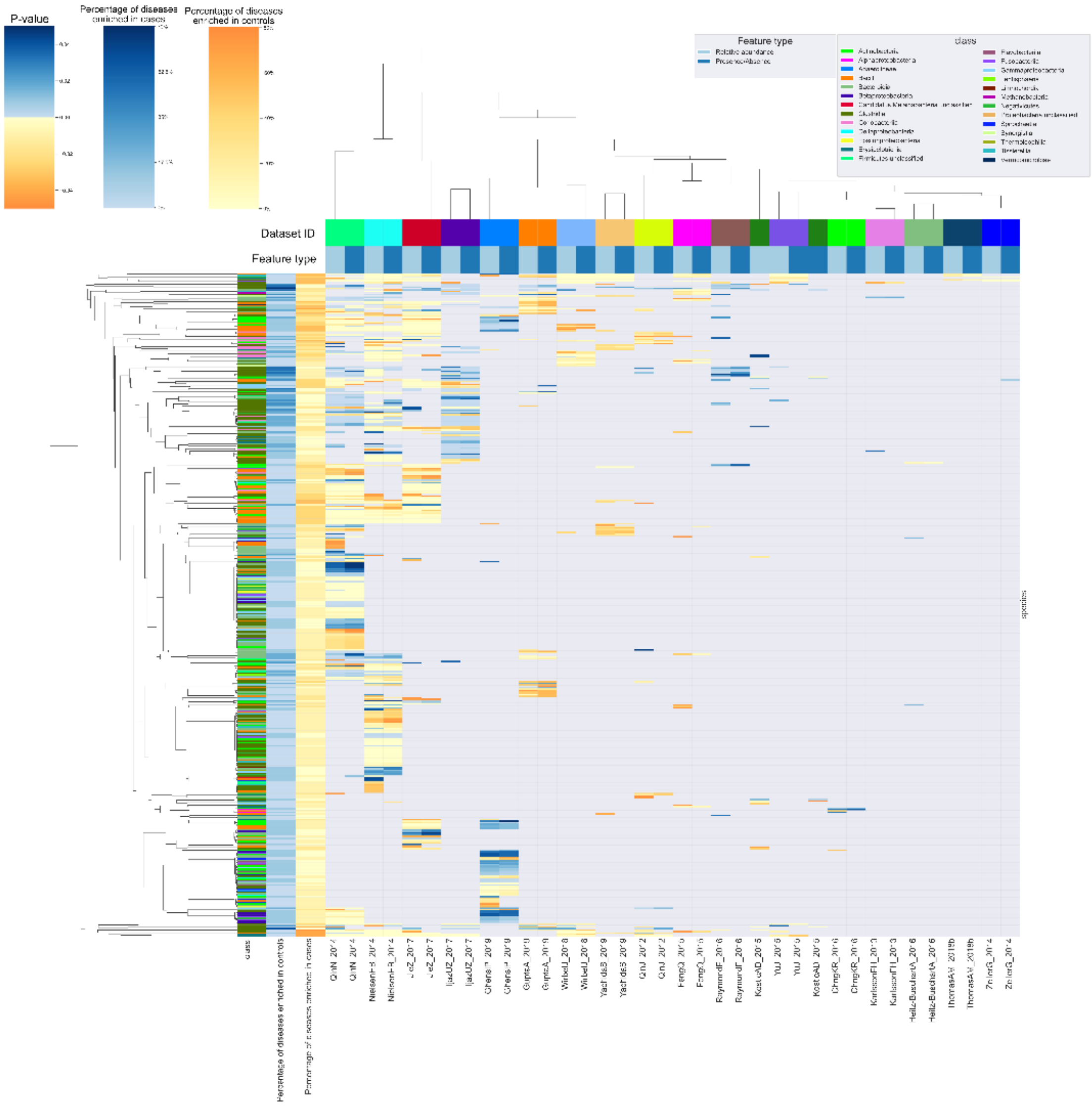


**S7 Fig.**

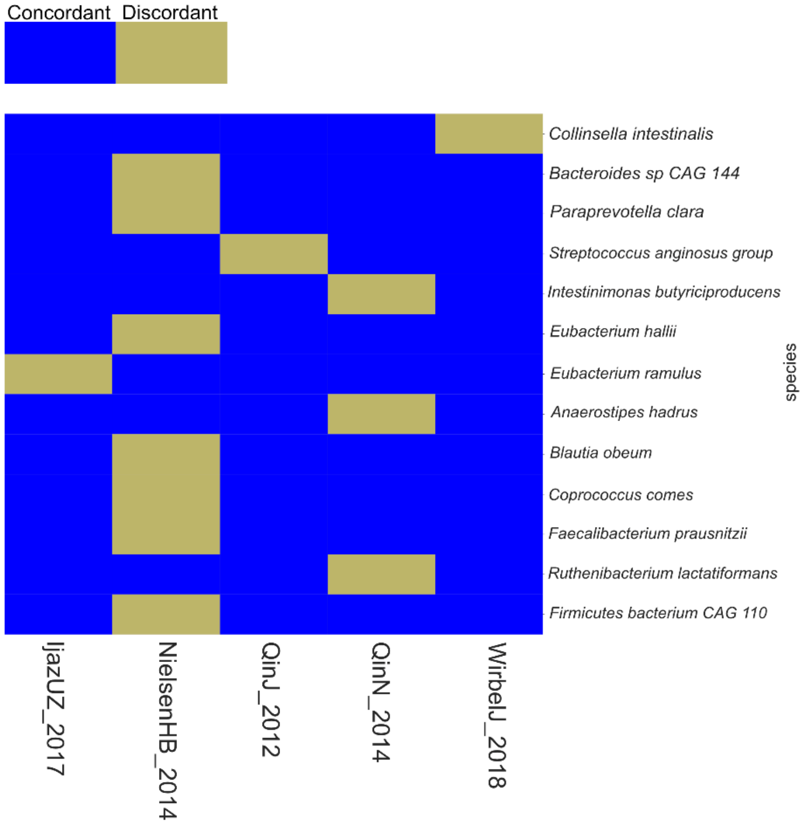


**S8 Fig.**

**We ask the authors to consider pointing to Figs. S1 and S2 from the caption of Fig 1.**

<u>Response</u>

We pointed the two supplementary figures in the caption of **Fig 1**:

**Fig 1**. **Classification accuracies are robust to degradation from species-level relative abundance to presence/absence profiles in shotgun datasets.** Results obtained on 25 case-control studies for host phenotype classification from human microbiomes. (**A**) Number of case and control samples across the different studies. (**B**) AUC and (**C**) AUPRC scores using RF as back-end classifiers on species-level taxonomic profiles. Comparison between relative abundance (in blue) and presence/absence (in red) profiles highlighted negligible differences and no statistical differences in none of the studies (see **S1 Fig** for AUC scores and **S2 Table** for p-values). Metrics of comparison in terms of AUC, AUPRC, precision, recall, and F1 are summarized in **S2 Table** and **S2 Fig**. (**D**) Number of statistically significant taxa from relative abundance (in blue) and presence/absence (in red) profiles.
